# Estimating virus effective population size and selection without neutral markers

**DOI:** 10.1371/journal.ppat.1006702

**Published:** 2017-11-20

**Authors:** Elsa Rousseau, Benoît Moury, Ludovic Mailleret, Rachid Senoussi, Alain Palloix, Vincent Simon, Sophie Valière, Frédéric Grognard, Frédéric Fabre

**Affiliations:** 1 Université Côte d’Azur, Inria, INRA, CNRS, UPMC Univ Paris 06, Biocore team, Sophia Antipolis, France; 2 Université Côte d’Azur, INRA, CNRS, ISA, Sophia Antipolis, France; 3 Pathologie Végétale, INRA, 84140 Montfavet, France; 4 UR BioSp, INRA, Avignon, France; 5 UR GAFL, INRA, Montfavet, France; 6 UMR BFP, INRA, Villenave d’Ornon, France; 7 GeT-PlaGe, INRA, Genotoul, Castanet-tolosan, France; 8 UAR DEPT GA, INRA, Castanet-Tolosan, France; 9 UMR SAVE, INRA, Villenave d’Ornon, France; Universidad Politecnica de Madrid, SPAIN

## Abstract

By combining high-throughput sequencing (HTS) with experimental evolution, we can observe the within-host dynamics of pathogen variants of biomedical or ecological interest. We studied the evolutionary dynamics of five variants of *Potato virus Y* (PVY) in 15 doubled-haploid lines of pepper. All plants were inoculated with the same mixture of virus variants and variant frequencies were determined by HTS in eight plants of each pepper line at each of six sampling dates. We developed a method for estimating the intensities of selection and genetic drift in a multi-allelic Wright-Fisher model, applicable whether these forces are strong or weak, and in the absence of neutral markers. This method requires variant frequency determination at several time points, in independent hosts. The parameters are the selection coefficients for each PVY variant and four effective population sizes *N*_*e*_ at different time-points of the experiment. Numerical simulations of asexual haploid Wright-Fisher populations subjected to contrasting genetic drift (*N*_*e*_ ∈ [10, 2000]) and selection (|*s*| ∈ [0, 0.15]) regimes were used to validate the method proposed. The experiment in closely related pepper host genotypes revealed that viruses experienced a considerable diversity of selection and genetic drift regimes. The resulting variant dynamics were accurately described by Wright-Fisher models. The fitness ranks of the variants were almost identical between host genotypes. By contrast, the dynamics of *N*_*e*_ were highly variable, although a bottleneck was often identified during the systemic movement of the virus. We demonstrated that, for a fixed initial PVY population, virus effective population size is a heritable trait in plants. These findings pave the way for the breeding of plant varieties exposing viruses to stronger genetic drift, thereby slowing virus adaptation.

## Author summary

A growing number of experimental evolution studies are using an “evolve-and-resequence” approach to observe the within-host dynamics of pathogen variants of biomedical or ecological interest. The resulting data are particularly appropriate for studying the effects of evolutionary forces, such as selection and genetic drift, on the emergence of new pathogen variants. However, it remains challenging to unravel the effects of selection and genetic drift in the absence of neutral markers, a situation frequently encountered for microbes, such as viruses, due to their small constrained genomes. Using such an approach on a plant virus, we observed that the same set of virus variants displayed highly diverse dynamics in closely related plant genotypes. We developed and validated a method that does not require neutral markers, for estimating selection coefficients and effective population sizes from these experimental evolution data. We found that the viruses experienced considerable diversity in genetic drift regimes, depending on host genotype. Importantly, genetic drift experienced by virus populations was shown to be a heritable plant trait. These findings pave the way for the breeding of plant varieties exposing viruses to strong genetic drift, thereby slowing virus adaptation.

## Introduction

Evolution in isolated populations results from the interplay between several forces, including mutation, selection, and genetic drift. Mutation creates genetic diversity within a population. Subsequent selection and genetic drift drive the evolution of diversity within the population. Selection is a deterministic force that increases the frequency of the fittest variants at the expense of the weakest ones. It can be characterized by the selection coefficient *s*, commonly calculated, at a specific locus, as the relative difference in fitness conferred by two alleles. Genetic drift, unlike selection, acts equally on all variants. It is the outcome of random sampling effects between generations, resulting in stochastic fluctuations in variant frequencies [1]. The strength of genetic drift is frequently evaluated by determining the effective population size *N*_*e*_ [1]. *N*_*e*_ is defined as the size of an ideal panmictic population of constant size with non-overlapping generations that would display the same degree of randomness in allele frequencies as the population studied [2]. *N*_*e*_ is often much lower than the census population size [3, 4], but it can be seen as its evolutionary analog [5]. When *N*_*e*_ is small, sampling effects are magnified between generations, and allele frequencies therefore fluctuate strongly. For populations varying in size over time, the effective population size over a given number of generations can be approximated by the harmonic mean ${\bar{N}}_{e}$ of effective population sizes at each generation. This approximation holds provided that the number of generations is much smaller than ${\bar{N}}_{e}$ [6–8] and that mutation can be neglected [9]. Population size may vary over time due to bottlenecks, which are common in natural populations. As they greatly decrease population size, they have a disproportionate effect on the overall value of ${\bar{N}}_{e}$ [1].

When selection and genetic drift act simultaneously, the probability of fixation of a new mutation (with a selection coefficient *s*), and, more generally, its evolutionary dynamics, is controlled by the product *N*_*e*_ × |*s*| [1, 10]. If *N*_*e*_ × |*s*| ≪ 1, then genetic drift predominates over selection and evolution is mostly stochastic. If *N*_*e*_ × |*s*| ≫ 1, then selection becomes effective and evolution is mostly deterministic [10]. This rule of thumb can be applied to the evolutionary dynamics of pathogen variants of biomedical or ecological interest, during the course of infection of a single host, for microbe variants escaping the immune response of their host, or becoming resistant to drug therapy (e.g. [11]) or, in the case of plant pathogens, for variants adapting to host resistance genes (e.g. [12]). In this study, we combined high-throughput sequencing (HTS) with experimental evolution to measure the within-host dynamics of five variants of *Potato virus Y* (PVY, genus *Potyvirus*, family Potyviridae) in closely related plant genotypes [13].

It remains challenging to unravel the effects of genetic drift and selection in the absence of neutral markers, in studies of the adaptation dynamics of pathogens. This situation is frequently encountered for pathogens with small genomes, especially viruses [14, 15]. Various approaches based on moment [16, 17] or likelihood [18–20] methods have been proposed for estimating *N*_*e*_, but all require the genetic markers studied to be neutral. Various methods have also been proposed for detecting selection and estimating selection coefficients. These methods require at least some prior information about *N*_*e*_ (e.g. [21]) or assume that genetic drift is negligible (e.g. [22]). However, in the absence of neutral markers and without prior estimates of *N*_*e*_, both selection and genetic drift must be taken into account, as these two forces act simultaneously on evolution. This greatly complicates the estimation of *N*_*e*_ and *s*. Only a few methods have been proposed for the joint estimation of *N*_*e*_ and *s* from time-sampled data (see [11] and [23] for a review). For large effective population sizes (typically *N*_*e*_ > 5000) and small selection coefficients (typically |*s*| < 0.01), several likelihood methods based on diffusion approximations of the Wright-Fisher model [1, 11] are available [24–27]. In the situations in which these methods are valid, the ranges of *N*_*e*_ and *s* values obtained are rather restrictive for many microorganisms, particularly viruses [28–31]. Foll *et al*. [32] recently proposed the use of approximate Bayesian computation (ABC) for the joint estimation of *N*_*e*_ and *s* in a Wright-Fisher model. Their method can deal with both weak and strong selection regimes, but still requires multilocus genome-wide data with mostly neutral loci to estimate *N*_*e*_ accurately.

In this study, we investigated the evolutionary dynamics of five variants of PVY in 15 closely related pepper genotypes. All plants were inoculated with the same mixture of virus variants and variant frequencies were determined with HTS in eight plants of each genotype at each of six sampling dates after inoculation. A diverse range of evolutionary patterns was observed. We developed a method for estimating the parameters of a multi-allelic Wright-Fisher model with selection and genetic drift, to investigate the underlying evolutionary processes. This method has two main advantages: it applies to a large range of selection and genetic drift intensities and it works efficiently in the absence of neutral markers. The parameters of the Wright-Fisher model (i.e. selection coefficients for each virus variant and effective population sizes at given time points) can be estimated by coupling maximum likelihood and ABC methods and applying them to a set of variant frequencies determined at several time points in independent hosts. We tested the method with numerical simulations mimicking the datasets obtained with HTS in evolve-and-resequence experiments [33]. The simulations covered an extensive range of *N*_*e*_ and *s* values. We were then able to estimate the selection coefficient of each PVY variant in each pepper genotype and the changes in effective population size over time during the colonization of the plant by the virus. Finally, by varying pepper genotypes and fixing the initial PVY population, we provided evidence that the effective population size of PVY is a heritable plant trait. This finding paves the way for the breeding of plant cultivars exposing viruses to greater genetic drift and/or smaller selection effects.

## Materials and methods

### Biological experiment

#### Plant and virus material

We used 15 doubled-haploid (DH) lines of pepper (*Capsicum annuum*, family Solanaceae). All the plants of a given genotype were thus genetically identical. All DH lines carried the major resistance gene *pvr2^3^* and differed in terms of their genetic background [12]. They are issued from the *F*_1_ hybrid between two pepper lines, Perennial and Yolo Wonder. Thus, on average, any pair of DH lines had 50% of alleles in common, at markers distinguishing between Perennial and Yolo Wonder. Each DH line therefore constituted a different host environment for plant colonization by PVY. These lines were chosen for study on the basis of quantitative differences in three previously measured factors, so as to generate different intensities of genetic drift and selection acting on PVY populations: (i) relative within-plant viral accumulation, (ii) resistance breakdown (RB) frequency [12] and (iii) the number of primary infection foci after mechanical inoculation with the virus [34] (S1 Fig).

**Fig 1:**
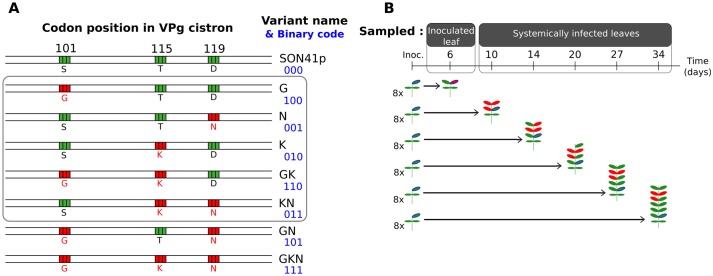
Virus variants inoculated to pepper plants and sampling protocol. (A) The five virus variants (in the gray box) were derived from the SON41p PVY clone and differed only at codon positions 101, 115 and 119 of the VPg cistron. These positions are shown in green if they correspond to the SON41p clone and in red if a non-synonymous substitution was introduced by site-directed mutagenesis. Single-letter amino acid abbreviations are presented below each position and PVY variant. Variant names and the corresponding binary code for the three point mutations of interest are given on the right of the sequences, with the binary code of the SON41p variant set to 000. The two additional possible variants, based on the three-digit binary code, are also shown at the bottom. (B) Sampling protocol for one pepper genotype. We inoculated 48 plants with the virus. Eight plants were sampled at each sampling time, from 6 to 34 days post-inoculation. The leaf circled in blue is the leaf inoculated with the virus. The leaves sampled are shown in red.

All plants were mechanically inoculated with the same equimolar mixture, based on quantitative double-antibody sandwich enzyme-linked immunosorbent assay (DAS-ELISA), of the five PVY variants G, N, K, GK and KN [35]. Single- and double-letter names indicate single and double mutants, respectively, of the infectious clone SON41p (Fig 1A). Three mutations located close together in the PVY genome differentiate the five variants, and these mutations are named after the amino-acid substitutions observed at positions 101 for the S (serine) to G (glycine) substitution, 115 for the T (threonine) to K (lysine) substitution, and 119 for the D (aspartic acid) to N (asparagine) substitution, in the VPg (viral protein genome-linked). The G and N variants displayed a low level of adaptation to the major resistance gene *pvr2^3^* carried by all plant genotypes, whereas variants K, GK and KN displayed higher levels of adaptation [12].

#### Experimental set-up and plant sampling

For each pepper genotype, 48 plants were arranged in randomized blocks, to minimize environmental effects. The first true leaf of each plant was inoculated 29 days after sowing. We then analyzed eight plants per DH line at 6, 10, 14, 20, 27 and 34 days post-inoculation (dpi) (Fig 1B). The inoculated leaf was sampled at 6 dpi, and, on subsequent sampling dates, three uninoculated leaves, corresponding to the three youngest unfolded leaves, were sampled and pooled together. As the plants were removed after sampling, the virus populations obtained from each plant sample were independent. This prevented possible effects on virus population dynamics due to the removal of infected leaves and subsequent re-sampling. Each leaf sample was ground in four volumes of 0.03 M phosphate buffer (pH 7.0) supplemented with 2% (w/v) diethyldithiocarbamate, as previously described [35].

#### High-throughput sequencing and determination of PVY variant frequencies

Total RNA was purified from individual plant samples with the Tri-Reagent kit (Sigma-Aldrich). It was subjected to reverse transcription-polymerase chain reaction (RT-PCR) with tagged primers for the amplification, over 35 cycles, of a 104-nucleotide region encompassing the polymorphic region of the PVY VPg cistron. Eight differently tagged primers were used, corresponding to the eight different plant replicates of the same plant genotype for each sampling date (S1 Table). Amplified DNAs corresponding to the eight plant replicates were pooled together on the basis of their intensity on electrophoresis gels.

HTS was performed at the Genomic Platform of INRA Toulouse. For this purpose, 2 × 150 base-pair (bp) libraries with multiplex adapters were prepared, and all the RT-PCR-amplified products were pooled into a single large sample (12 cycles). This sample was run on a MiSeq Illumina paired-end sequencer with the MiSeq Reagent Kit v2, for 500 cycles. We chose to use MiSeq Illumina sequencing because this technology has a much lower error rate than other high-throughput sequencing technologies, such as 454 sequencing [36]. By using tagged primers and subsequent multiplex adapters, we were able to assign a plant genotype and a sampling date to each sequence.

In the initial sequence analysis, we used FLASH software to obtain the consensus sequence from reads 1 and 2 with a minimum overlap length between the two reads of 63, a maximum overlap length of 153 and a maximum allowed ratio of the number of mismatched base pairs to overlap length of 0.2 [37]. The sequences were then sorted by adapter and by tag. Finally, the sequences corresponding to each PVY variant were determined with the help of ‘agrep’ function in R software [38], and sequence counts were used to assess the composition of the virus population in each sample. After sequence sorting, we had 374 to 14141 sequences per sample, with a mean of 3295 sequences per sample.

We carried out two complementary sequence analyses to detect PVY mutations (see S1 Text for details). It was important to perform these analyses as the presence of mutants might have affected virus population dynamics and the intensities of the evolutionary forces studied. In the first analysis, we looked for all eight possible variants based on the three codon positions of interest in the VPg cistron, *i.e.* the five analyzed variants, G, N, K, GK and KN, together with the SON41p, GN and GKN variants (corresponding to all the possible binary codes in Fig 1A). The sum of the frequencies of all three additional variants, SON41p, GN and GKN, remained below 5%, and these variants were not, therefore, considered in *N*_*e*_ and *s* estimations. The raw data from this analysis (*i.e.* number of sequences of each variant in each of the 677 samples analyzed) are available from S2 Table. We then calculated the frequencies of *de novo* substitutions in each sample and at each nucleotide position of all sequences, by comparison with the sequence of the SON41p reference clone (equivalent to comparison with sequences of the G, N, K, GK and KN clones). In all, only PVY populations sampled from six of the 677 plants studied presented a *de novo* substitution with a frequency exceeding 5% (S1 Text). These PVY populations were removed for subsequent analyses. Furthermore, numerical simulations showed that a sixth unaccounted for variant present at a mean frequency of 7% in virus populations had no significant impact on estimates of *N*_*e*_ and *s* (see below), justifying our use of a 5% threshold.

#### Genetic analysis and heritability estimation

With this experimental design, we studied the phenotype of each pepper DH line in terms of its effect on PVY populations. The plants within each pepper DH line were genetically identical and experimental conditions were set up so as to ensure an absence of differences in environmental effects between DH lines. We were therefore able to estimate the heritability of plant traits of interest, corresponding to the evolutionary forces exerted by the plant on PVY populations, by constituting two replicates for each DH line dataset. More precisely, we assessed the heritability of the intrinsic rates of increase for the five PVY variants and of the effective population sizes of PVY. As the initial population of PVY was fixed and identical for all plant genotypes, we did not consider the effect of PVY population composition on the heritability of effective PVY population size. To estimate heritabilities, we split the dataset for the 48 plants for each DH line in two, by randomly selecting four of the eight plants at each sampling date. The broad-sense heritability of any plant trait of interest can be estimated as $h^{2}=\sigma_{G}^{2}/\left(\sigma_{G}^{2}+\sigma_{e}^{2}/n\right)$, where $\sigma_{G}^{2}$ corresponds to the genotypic variance, $\sigma_{e}^{2}$ to the phenotypic variance and *n* to the number of replicates [39], the variance being set to the sum of the squared deviations from the mean.

### Estimation of selection and genetic drift intensities

We developed a method for estimating the parameters of a multi-allelic Wright-Fisher model with selection and genetic drift for a haploid population. The parameters and state variables of the model and the observed variables are summarized in Table 1.

**Table 1:** Main notations for the observations and the model.

	Designation (unit) [reference value]
**Observed variables**	
$x^{p}\left(t_{d}\right)=\left(x_{1}^{p}\left(t_{d}\right),\ldots ,x_{n_{var}}^{p}\left(t_{d}\right)\right)$	Variant sequence counts in virus population *p* at sampling time *t*_*d*_ (*seq* ^a^)
$f^{p}\left(t_{d}\right)=\left(f_{1}^{p}\left(t_{d}\right),\ldots ,f_{n_{var}}^{p}\left(t_{d}\right)\right)$	Variant frequencies in virus population *p* at sampling time *t*_*d*_ (no unit)
**State variables**	
$\lambda^{p}\left(t\right)=\left(\lambda_{1}^{p}\left(t\right),\ldots ,\lambda_{n_{var}}^{p}\left(t\right)\right)$	Theoretical variant frequencies in virus population *p* at time *t* of a Wright-Fisher model (no unit)
**Parameters of interest**	
$r=(r_{1},\ldots ,r_{n_{var}})$	Variant relative intrinsic rates of increase (*generation*^−1^) ^a^
$\eta_{e}=(\eta_{e}^{IO},\eta_{e}^{S_{1}},\eta_{e}^{S_{2}},\eta_{e}^{S_{3}})$	Successive virus effective population sizes (*individuals*) ^b^
**Fixed parameters**	
**λ**^*inoc*^	Vector of variant frequencies in the virus inoculum (no unit)
***T***	Vector of measurement dates (*day*) [(0, 6, 10, 14, 20, 27, 34)]
**Additional notations**	
***N*_*e*_** = (*N*_*e*_(1), …, *N*_*e*_(34))	Vector of virus effective population sizes (piecewise constant function of *η*_*e*_)
$N_{e}^{h}\left(t_{d}\right)$	Harmonic mean of virus effective population sizes at sampling time *t*_*d*_
$\sigma [f_{i}^{•}\left(t_{d}\right)]$	Standard deviation of the frequencies of virus variant *i* at sampling time *t*_*d*_ over the virus populations *p*
$\lambda^{det}(t)=(\lambda_{1}^{det}(t),\ldots ,\lambda_{n_{var}}^{det}(t))$	Vector of variant frequencies at time *t* for an infinite size Wright-Fisher model

^a^ The abbreviation “seq” is the number of sequences representing the virus population or a given variant in this population.

^b^ The mean intrinsic rate of increase $\bar{r}$ of all virus variants is one.

^c^ With the full model $M_{4}$, $\eta_{e}^{IO}$ in the inoculated organ for *t* ∈ [1, 6], $\eta_{e}^{S1}$ at the onset of systemic infection for *t* ∈ [7, 10], $\eta_{e}^{S2}$ for *t* ∈ [11, 14] and $\eta_{e}^{S3}$ for *t* ∈ [15, 34].

#### Notation: Observed variables, state variables and parameters of interest

**λ**^*inoc*^ denotes the vector of the observed variant frequencies in the parental virus population, i.e. the inoculum used to inoculate all host plants in the experiment. Thereafter, the measurement date vector ***T*** = (0, 6, 10, 14, 20, 27, 34) is indexed by *t*_*d*_ (*d* = 0, …, 6). In particular, *t*_0_ = 0 is the inoculation date and *t*_*d*_ (*d* = 1, …, 6) are the sampling dates. The time, indexed by *t* = 1, …, 34, is the number of days post-inoculation and indicates the viral generation, as the generation time was assumed to be one day [40]. For a given plant genotype, at each sampling date *t*_*d*_, a sample of *n*_*inf*_(*t*_*d*_) infected plants was observed, for which we measured the vectors $x^{p}\left(t_{d}\right)=\left(x_{1}^{p}\left(t_{d}\right),\ldots ,x_{n_{var}}^{p}\left(t_{d}\right)\right)$, with $x_{i}^{p}\left(t_{d}\right)$ the number of sequences obtained for virus variant *i* (1 ≤ *i* ≤ *n*_*var*_) in population *p* (1 ≤ *p* ≤ *n*_*inf*_(*t*_*d*_)). Thereafter, replacing an index in a notation by • is equivalent to summing over the corresponding index set. The total number of sequences obtained from virus population *p* at time *t*_*d*_ is thus $x_{•}^{p}\left(t_{d}\right)$. Finally, $f^{p}\left(t_{d}\right)=\left(f_{1}^{p}\left(t_{d}\right),\ldots ,f_{n_{var}}^{p}\left(t_{d}\right)\right)$, with $f_{i}^{p}\left(t_{d}\right)$ the observed frequency of virus variant *i* in population *p* at sampling date *t*_*d*_. It is calculated as $x^{p}\left(t_{d}\right)/x_{•}^{p}\left(t_{d}\right)$.

The state variable of interest is $\lambda^{p}\left(t\right)=\left(\lambda_{1}^{p}\left(t\right),\ldots ,\lambda_{n_{var}}^{p}\left(t\right)\right)$, with $\lambda_{i}^{p}\left(t\right)$ the frequency of virus variant *i* (1 ≤ *i* ≤ *n*_*var*_) in virus population *p* at any date *t* = 1, …, 34. Virus variant dynamics are represented by a Wright-Fisher model (see below) to infer ***θ*** = (***r***, ***η***_*e*_), the vector of parameters describing the underlying evolutionary forces. $r=(r_{1},\ldots ,r_{n_{var}})$ is the vector of the intrinsic rates of increase *r*_*i*_ of each virus variant *i*. We assumed that the mean intrinsic rate of increase $\bar{r}$ over all variants was one, as we were interested only in the relative intrinsic rates of increase. The selection coefficient of a variant *i* is usually computed as *s*_*i*_ = *r*_*i*_ − 1. The vector parameter ***η***_*e*_ defines a piecewise function describing effective population sizes *N*_*e*_(*t*). We determined the temporal variation of effective population sizes, using four models with ***η***_*e*_ having one to four parameters. With the more general model $M_{4}$, $\eta_{e}=(\eta_{e}^{IO},\eta_{e}^{S_{1}},\eta_{e}^{S_{2}},\eta_{e}^{S_{3}})$. $\eta_{e}^{IO}$ is the effective population size of the viral population in the inoculated organ; this stage lasts *t*_1_ = 6 days in our experimental design. $\eta_{e}^{S_{1}}$ is the effective population size during the onset of systemic infection; this stage lasts *t*_2_ − *t*_1_ = 4 days. $\eta_{e}^{S_{2}}$ is the effective population size during the next *t*_3_ − *t*_2_ = 4 days and $\eta_{e}^{S_{3}}$ the effective population size later on, during the last *t*_6_ − *t*_3_ = 20 days of survey. Accordingly, we define *N*_*e*_(*t*) as follows:

$$\begin{array}{c} N_{e}\left(t\right)=\{\begin{array}{cc} \eta_{e}^{IO} & \;t\in [1,\ldots ,t_{1}]\\ \eta_{e}^{S_{1}} & \;t\in [t_{1}+1,\ldots ,t_{2}]\\ \eta_{e}^{S_{2}} & \;t\in [t_{2}+1,\ldots ,t_{3}]\\ \eta_{e}^{S_{3}} & \;t\in [t_{3}+1,\ldots ,t_{6}] \end{array} \end{array}$$ (1)

With model $M_{3}$, $\eta_{e}=(\eta_{e}^{IO},\eta_{e}^{S_{1}},\eta_{e}^{S_{2}})$. *N*_*e*_(*t*) has three parameters: (i) $N_{e}\left(t\right)=\eta_{e}^{IO}$ when *t* ∈ [1, 6], (ii) $N_{e}\left(t\right)=\eta_{e}^{S_{1}}$ when *t* ∈ [7, 14] and (iii) $N_{e}\left(t\right)=\eta_{e}^{S_{2}}$ when *t* ∈ [15, 34]. With model $M_{2}$, $\eta_{e}=(\eta_{e}^{IO},\eta_{e}^{S})$. *N*_*e*_(*t*) has two parameters: (i) $N_{e}\left(t\right)=\eta_{e}^{IO}$ when *t* ∈ [1, 6] and (ii) $N_{e}\left(t\right)=\eta_{e}^{S}$ when *t* ∈ [7, 34]. Finally, with model $M_{1}$, ***η***_*e*_ = (*η*_*e*_): the effective population size for the virus remains constant throughout the experiment (*N*_*e*_(*t*) = *η*_*e*_ for *t* ∈ [1, 34]). The effective population size at any sampling date of interest *t*_*d*_ is given, approximately, by the harmonic mean of the effective sizes of the successive generations $N_{e}^{h}\left(t_{d}\right)={\left(\frac{1}{t_{d}}\sum_{j=1}^{t_{d}}\frac{1}{N_{e}\left(j\right)}\right)}^{-1}$.

#### The multi-allelic Wright-Fisher model with selection and genetic drift

The Wright-Fisher model occupies a central position in population genetics [41]. It assumes an ideal population: a randomly mating haploid population of finite size reproducing in discrete non-overlapping generations, with no structure. By definition, *N*_*e*_ is the size of an ideal population (i.e. obeying previous assumptions) that would display the same degree of randomness in variant frequencies as the real population studied [2]. As for any model-based approach, the use of this concept requires the actual population being not too far from an ideal Wright-Fisher model with suitable parameters [10]. Formally, the Wright-Fisher model is very similar to the quasispecies model describing the evolution of DNA (or RNA) sequences in finite populations [42]. In practice, the Wright-Fisher model has been used to infer the evolutionary history of viruses (e.g. [11]) and it can also be used to describe the stochastic dynamics of the frequency of the *n*_*var*_ virus variants considered here. Let ***z***(*t*) be the vector of the number of each virus variant *i* (1 ≤ *i* ≤ *n*_*var*_) in generation *t* and, with previous notations, let $\lambda \left(t\right)=\frac{z(t)}{N_{e}\left(t\right)}$ be the corresponding vector of variant frequencies. The dynamics of ***z***(*t*) are shaped by random genetic drift and selection. Let $pr\left(t\right)=(pr_{1}\left(t\right),\ldots ,pr_{n_{var}}\left(t\right))$ be the vector of the probabilities of sampling each virus variant from generation *t* to generation *t* + 1. For *t* ≥ 1, the distribution of ***z***(*t* + 1) conditionally on ***z***(*t*) follows a multinomial distribution [41]:

$$\{\begin{array}{ll} z(t+1)|z(t)\sim \text{Mult}(\text{size}=N_{e}(t),\text{prob}=pr(t)) & \left(2\right)\\ pr_{i}(t)=\frac{r_{i}\lambda_{i}(t)}{\sum_{j=1}^{n_{var}}r_{j}\lambda_{j}(t)}\;\text{with}\;\lambda_{i}(t)=\frac{z_{i}(t)}{N_{e}(t)}\;\text{and}\;\lambda (0)=\lambda^{inoc} & \left(3\right) \end{array}$$

In the model, selection reweights the different genotypes according to their constant fitness. Fitness does not depend on the composition of the population (*i.e.* there is no frequency-dependent selection). As population size tends to ∞ (*i.e.* genetic drift becomes negligible), the stochastic process of variant frequencies described by eq (2) converges on deterministic recursion describing $\lambda_{i}^{det}\left(t\right)$ which approximates the mean frequency of variant *i* at generation *t*. For *t* ≥ 1:

$$\begin{array}{c} \lambda_{i}^{det}\left(t+1\right)|r=\frac{r_{i}\lambda_{i}^{det}\left(t\right)}{\sum_{j=1}^{n_{var}}r_{j}\lambda_{j}^{det}\left(t\right)}\;\text{with}\;\lambda^{det}\left(0\right)=\lambda^{inoc} \end{array}$$ (4)

#### Parameter estimation

We propose an approach combining a first step relying on maximum-likelihood followed by a step relying on ABC, to estimate the parameters of interest ***θ*** = (***r***, ***η***_*e*_). The first step estimates the vector of the relative intrinsic rates of increase of each virus variant ***r*** by maximum-likelihood methods. Let the vector $x^{•}\left(t_{d}\right)=\left(x_{1}^{•}\left(t_{d}\right),\ldots ,x_{n_{var}}^{•}\left(t_{d}\right)\right)$ be the total number of sequences of virus variant *i* obtained in the *n*_*inf*_(*t*_*d*_) infected plants (of a given plant genotype) at sampling date *t*_*d*_. This step assumes that $x^{•}\left(t_{d}\right)∼Mult(\text{size}=\sum_{i=1}^{n_{var}}x_{i}^{•}\left(t_{d}\right),\text{prob}=\lambda^{det}\left(t_{d}\right)|r)$. Let ***x*** denote the vector of all total sequence counts, at all sampling time-points, constituting one dataset. As the samples are independent between sampling dates, the likelihood function is:

$$\begin{array}{c} l\left(x|r\right)=\prod_{d=1}^{6}dM(\text{size}=\sum_{i=1}^{n_{var}}x_{i}^{•}\left(t_{d}\right),\text{prob}=\lambda^{det}\left(t_{d}\right)|r), \end{array}$$

*dM* being the probability density function (pdf) of the multinomial distribution. Under these hypotheses, ***r*** can be inferred by minimizing −*log*(*l*(***x***|***r***)), assuming that the mean intrinsic rate of increase $\bar{r}$ of all variants is one. The estimate of ***r***, denoted $\hat{r}$, was obtained straightforwardly, using the ‘nlminb’ optimization routine implemented in R software version 3.0.2 [38].

The second step estimates the vector of effective population sizes ***η***_*e*_ of a given model $M_{j}$ (*j* = 1, ⋯, 4) with ABC, conditionally to $\hat{r}$. All ABC algorithms involve the simulation of a large number of possible datasets by sampling the parameters of interest (here ***η***_*e*_) from prior probability distributions *π*(***η***_*e*_). We used independent log-uniform priors on [10, 2500] for each parameter of ***η***_*e*_. For a given $\eta_{e}^{sim}$ sampled in *π*(***η***_*e*_), a dataset was simulated as follows. The first step was to simulate 48 (= 6 sampling dates * 8 plants/date) independent dynamics of evolution of virus variant frequencies lasting 34 days (max(***T***)) with the Wright-Fisher model (eqs 1, 2 and 3) parameterized by $\left(\hat{r},\eta_{e}^{sim}\right)$. Let $\lambda_{sim}^{l}\left(t\right)$ (*l* = 1, …, 48; *t* = 1, …, 34) be this set of simulated dynamics. The second step was to simulate the experimental design. Eight plants were analyzed with HTS at each sampling date, $x_{sim}^{tot,l}$ being the total number of sequences obtained for plant *l*. Variant counts $x_{sim}^{l}\left(t_{d}\right)$ for HTS are sampled from multinomial distributions. A single sample was obtained for each dynamic *l*: at 6 dpi, $x_{sim}^{l}\left(6\right)∼\text{Mult}(\text{size}=x_{sim}^{tot,l},\text{prob}=\lambda_{sim}^{l}\left(6\right))$ with *l* ∈ [1, 8]; at 10 dpi, $x_{sim}^{l}\left(10\right)$ is obtained similarly with *l* ∈ [9, 16], and so on, until 34 dpi, with *l* ∈ [41, 48]. The observed frequencies are $f_{sim}^{l}\left(t_{d}\right)=\frac{x_{sim}^{l}\left(t_{d}\right)}{x_{sim}^{tot,l}}$. The last step was to calculate the vector of summary statistics from the simulated dataset, $S_{sim}=(S_{sim}^{t_{1}},\ldots ,S_{sim}^{t_{6}})$. A single summary statistic was calculated for each sampling date. This statistic is the inverse of the mean of the standard deviation of the variant frequencies at sampling date *t*_*d*_. Formally, $S_{sim}^{t_{d}}={\left(\frac{1}{n_{var}}\sum_{i=1}^{n_{var}}\sigma \left[f_{sim,i}^{•}\left(t_{d}\right)\right]\right)}^{-1}$ where $\sigma [f_{sim,i}^{•}\left(t_{d}\right)]$ is the standard deviation (over the infected hosts at sampling date *t*_*d*_) of $f_{sim,i}^{l}\left(t_{d}\right)$, the observed frequency of variant *i*. In practice, estimation was performed with the adaptive ABC algorithm of Lenormand *et al.* [43] implemented in the R package EasyABC with tuning parameters *nb*_*simul*_ = 5000, *p*_*accmin*_ = 0.04 and *α* = 0.5. Models $M_{1}$, $M_{2}$, $M_{3}$ and $M_{4}$ (embedding *N*_*e*_(*t*) functions with one to four parameters) were compared, using the multinomial logistic regression method implemented in the ABC package (function postpr with 2.10^5^, 2.5 × 10^5^, 7.5 × 10^5^ and 1.5 × 10^6^ simulated summary statistics under models $M_{1}$, $M_{2}$, $M_{3}$ and $M_{4}$, respectively, and tuning parameter tol = 5 × 10^−4^). The estimation code will be made available upon request.

### Numerical simulations

Before using the estimation method on the datasets corresponding to the biological experiment, we performed several batches of simulations to assess its ability to infer effective population sizes and selection coefficients accurately (see S2 Text for details). Briefly, in experiment 1, we first simulated the changes in frequencies of five virus variants under 750 selection and genetic drift regimes with a Wright-Fisher model for haploid individuals. The simulations were designed to fit the experimental setup of our datasets (48 independent host plants regularly analyzed at 6 sampling dates). For each of the 750 datasets obtained, the true parameters ***θ***_*true*_ were known and could be compared with the estimated parameters $\hat{\theta }$. In experiment 2, we assessed the sensitivity of the estimation method to the presence of a sixth undetected virus variant. This sixth variant was selectively neutral (its selection coefficient is zero), present in the inoculum at a frequency of 3% and still present at the last sampling date (34 dpi) in all plants analyzed, at frequencies ranging from 1% to 6%. It affected the dynamics of the five variants of interest in all plants but was not detected, so the variant frequencies measured by HTS (and used to estimate $\hat{\theta }$) are noisy with respect to their true values. In all, 350 simulated datasets were analyzed in this second test.

## Results

In this section, we will (i) describe the virus dynamics observed in the biological experiment with 15 plant genotypes, (ii) validate the method for estimating selection and genetic drift with numerical simulations and (iii) describe the estimates obtained in the biological experiment.

### Virus variant dynamics in the 15 plant genotypes

**Fig 2:**
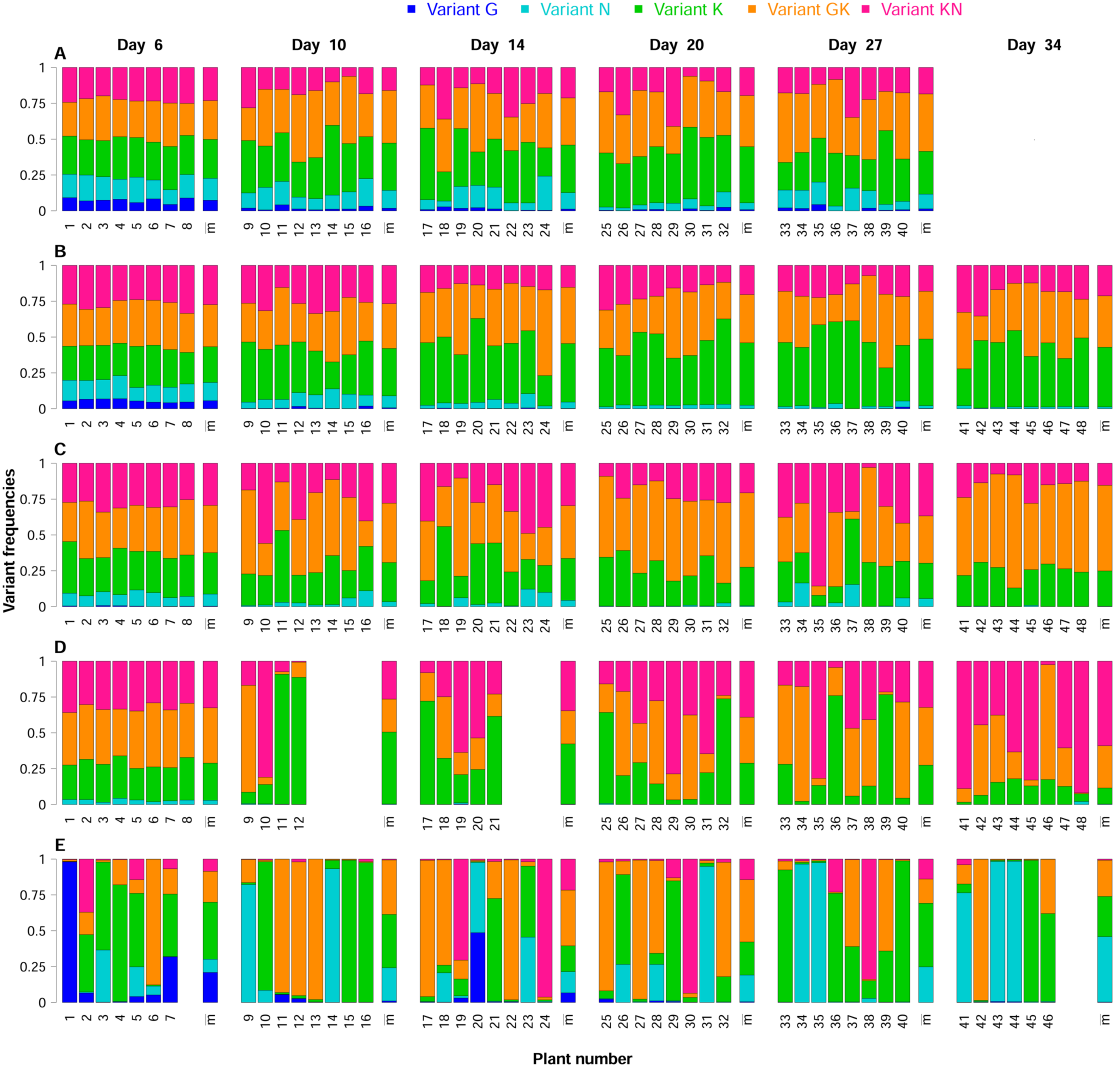
Five contrasting datasets obtained in the biological experiment. Each line of bar plots represents the dynamics of virus variants in a single DH line over time: (A) 240, (B) 2430, (C) 2344, (D) 2321 and (E) 219. We inoculated 48 plants per DH line, and we sampled eight plants, which were subsequently removed from the experiment, at each of the six sampling dates (6, 10, 14, 20, 27 and 34 days post-inoculation). Within each bar plot, the frequencies of the five variants (see top of the figure for the color code) in each infected plant sample are represented by single bars (labeled from 1 to 48). The missing bars correspond to plant samples for which no viruses were detected. The last bar indicates the mean viral composition in the infected plants. Each individual bar plot corresponds to a single sampling date, indicated at the top of each column of barplots. The five DH lines displayed contrasting virus variant dynamics, consistent with contrasting patterns of selection and genetic drift. We could not sample plants of DH line 240 (A) 34 days post-inoculation, because severe necrosis symptoms invading the stem led to the death of all plants at this sampling date.

The frequencies of the five virus variants were assessed in completely isolated populations during the course of infection, in 15 different plant genotypes. For each of these 15 pepper genotypes, 48 plants were inoculated with the same equimolar mixture of the five variants, and the frequencies of the virus variants were determined in eight plants at each of six sampling dates, from 6 to 34 days post-inoculation (Fig 2). In a few cases, no viruses were detected in plant samples (lacking bars in Fig 2). These negative samples may reflect the presence of an extreme bottleneck at inoculation, leading to virus population extinction, or a long time lag to systemic infection of the plant (for measurements from 10 to 34 dpi), resulting in the sampling of leaves not yet infected (e.g. DH line 2321). Negative samples were most frequent for the first two dates on which systemically infected leaves were analyzed, *i.e.* at 10 and 14 dpi, probably indicating a time lag to systemic infection in some DH lines. Negative samples were observed in only four DH lines (e.g. DH lines 219 and 2321). No infection was observed in a mean of 3.5 (resp. 2.0) plant samples 10 (resp. 14) dpi for the four DH lines concerned.

The virus populations present in all infected plants and in the common inoculum were analyzed by HTS, to determine the frequencies of the five PVY variants. Inoculum analysis confirmed that all variants were present in roughly equimolar proportions, with 22.6% of variant G, 17.5% of N, 20.6% of K, 17.1% of GK and 22.2% of KN.

The raw data for variant frequency dynamics provided considerably different patterns between the 15 pepper genotypes (Fig 2, S2 and S3 Figs). Variant frequencies were similar between virus populations sampled on the same date in some plant genotypes, consistent with weak genetic drift (e.g. DH lines 240 and 2430, Fig 2A and 2B), whereas they differed in other plant genotypes (e.g. DH lines 2321 and 219, Fig 2D and 2E). Furthermore, the heterogeneity of variant frequencies between the eight plants analyzed fluctuated between dates, probably due to changes in effective population size during the course of infection (e.g. DH line 2344, Fig 2C). The four pepper genotypes for which some samples were virus-negative were also characterized by the highest heterogeneity in variant frequencies, consistent with an extreme bottleneck at inoculation and/or during systemic movement of the virus (see DH lines 2321, 219, 2256 and 2400 in Fig 2D and 2E, S2D and S3I Figs). Selection regimes also differed between lines. In some DH lines, all variants remained present at all dates (e.g. DH line 240, Fig 2A), whereas one variant (e.g. DH line 219, Fig 2E), or up to two variants (e.g. DH lines 2430, 2344, 2321, Fig 2B–2D) became extinct in others.

### Validation of the estimation method with numerical simulations

Before its application to the experimental dataset, we validated the estimation method proposed by numerical simulations of a Wright-Fisher model with selection and genetic drift for haploid individuals.

#### Range of selection and genetic drift intensities explored

**Fig 3:**
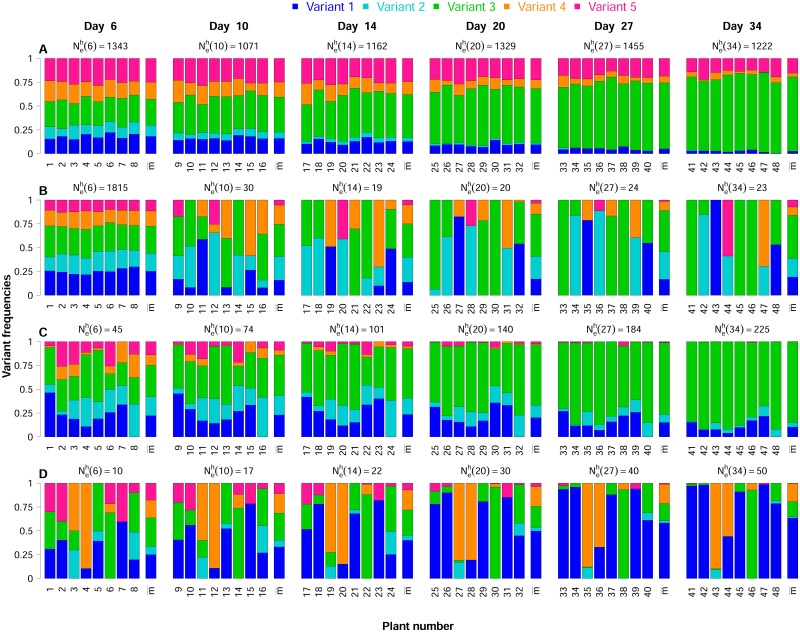
Contrasting datasets obtained in numerical experiment 1. For each dataset (series A to D), the composition of eight populations was observed at six sampling dates, from 6 to 34 days post-inoculation, in independently sampled hosts. Within each plot, each bar represents the composition of the population in one plant at one date, and the last bar shows the mean frequencies over these populations. The color code at the top is used to distinguish the five variants. The harmonic mean of effective population size is indicated in the main title of each plot. The parameter values used for the simulations are: series (A) ***r*** = (0.971, 0.92, 1.09, 0.992, 1.027), $N_{e}^{IO}=1343$, $N_{e}^{S_{1}}=822$; series (B) ***r*** = (1.05, 1.005, 1.077, 0.963, 0.904), $N_{e}^{IO}=1815$, $N_{e}^{S_{1}}=12$; series (C) ***r*** = (1.045, 1.031, 1.12, 0.879, 0.924), $N_{e}^{IO}=45$, $N_{e}^{S_{1}}=1473$; series (D) ***r*** = (1.105, 0.943, 0.999, 1.041, 0.912), $N_{e}^{IO}=10$, $N_{e}^{S_{1}}=1025$. Note that $N_{e}^{S_{1}}$ is used for the iterative computation of a sequence of effective population sizes varying each five generations during the systemic infection stage.

The 750 datasets generated in experiment 1 corresponded to very different selection and genetic drift regimes (S4 Fig). Randomly sampled effective population sizes in the inoculated organ and at the onset of systemic infection were combined independently, to simulate highly diverse dynamics of effective population size (see S2 Text). This led to the exploration of a large range of harmonic means of effective population size $N_{e}^{h}\left(t_{d}\right)$, ranging from 10 to 1996 (5% quantile = 22, mean = 230, 95% quantile = 873). Relative fitness values (*r*_*i*_) ranged from 0.75 to 1.27, independently of effective population size (S4 Fig). They reflect a mean absolute selection coefficient |*s*| of 0.08 (5% quantile = 0.007, 95% quantile = 0.18). As a result, highly diverse simulated datasets were obtained. Overall, the patterns encountered in the experimental datasets (Fig 2) were similar to some of those obtained for the simulated datasets. The simulated datasets also included a number of datasets with more extreme patterns of selection and genetic drift regimes. We illustrate the differences in the genetic drift regimes observed in Fig 3. For a given dataset, variant frequencies could be roughly similar (Fig 3A) or very different (Fig 3D) between populations, at all sampling dates. Moreover, the independent sampling of effective population sizes in the inoculated organ and during systemic infection generated genetic drift regimes that varied over time. For example, we observed strong similarities between populations at the first sampling date, but greater heterogeneity at subsequent dates (Fig 3B) and the opposite pattern (Fig 3C).

#### Parameter estimation accuracy

Effective population sizes $N_{e}^{h}\left(t_{d}\right)$ and intrinsic rates of increase of each variant *r*_*i*_ were inferred for each of the 750 datasets simulated in experiment 1 (with 5 virus variants) and the 350 datasets simulated in experiment 2 (with the 5 virus variants and an additional undetected sixth variant) using the more general model $M_{4}$. True parameters (*i.e.* known parameter values used in the simulations) and estimated parameter values were compared, to assess estimation accuracy.

**Fig 4:**
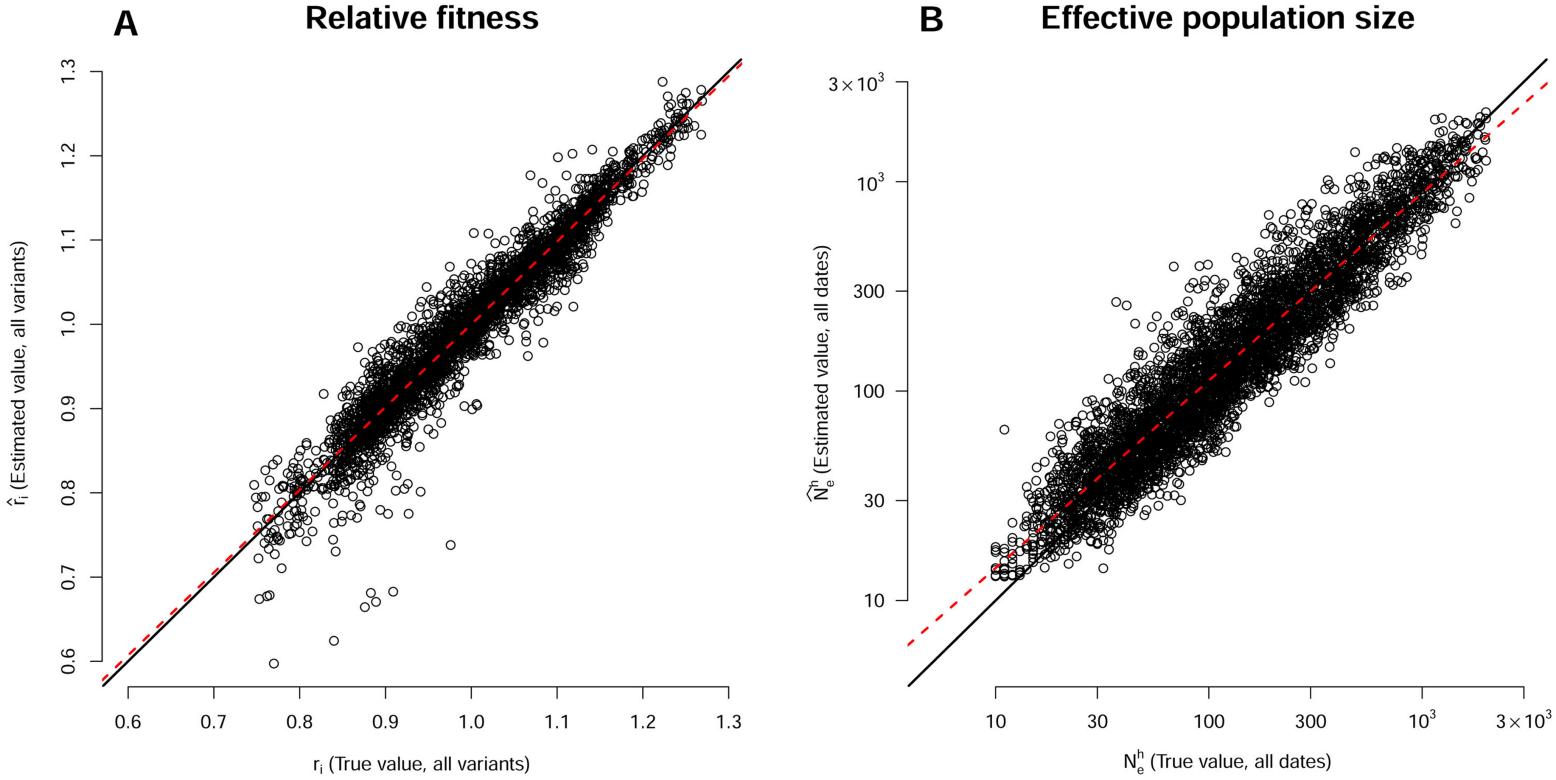
Inferences for variant fitness *r* and for the harmonic mean of effective population size $N_{e}^{h}\left(t_{d}\right)$, for the 750 datasets simulated with five virus variants. (A) Correlation between true *r*_*i*_ (x-axis) and estimated ${\hat{r}}_{i}$ (y-axis) (all variants considered together). (B) Correlation between true $N_{e}^{h}$ (x-axis) and estimated ${\hat{N}}_{e}^{h}$ (y-axis) (all sampling dates considered together, logarithmic scale). In both panels, the black line is the first bisector and the red dashed line is the best-fitting linear model. In panel A, the 9 points with ${\hat{r}}_{i}<0.7$ correspond to datasets in which a highly counterselected variant was observed in only a few plants (5, on average, of the 48 plants) due to an initial low effective population size.

In numerical experiment 1, HTS analysis provided samples of the true frequencies of the virus variants in the simulated Wright-Fisher populations. The estimates of the intrinsic rates of increase ${\hat{r}}_{i}$ were very accurate, with an *R*^2^ of the best-fit line of 0.93, a slope close to 1 (0.98) and an intercept of 0.02 (Table 2, Fig 4A). The estimates of the harmonic mean of the effective population size ${\hat{N}}_{e}^{h}\left(t_{d}\right)$ were also accurate, with a best-fit line close to the first bisector (Table 2, Fig 4B) (*R*^2^ = 0.86), despite a slight trend towards overestimation (slope = 0.91, intercept = 28). In both cases, mean relative bias was small and its 95% confidence interval included zero. The 90% confidence intervals of all estimated parameters were highly accurate. They included the true parameter values in nearly 90% (resp. 91%) of the cases for ${\hat{N}}_{e}^{h}\left(t_{d}\right)$ (resp. ${\hat{r}}_{i}$) (Table 2).

**Table 2:** Performance of the estimators of the harmonic mean of effective population sizes $N_{e}^{h}\left(t_{d}\right)$ and variant fitness *r* obtained with the two numerical experiments.

Experiment^a^	Parameter^b^	*R*^2^	Intercept	Slope	Accuracy of 90% CI	Mean bias [95% CI]
Experiment 1	${\hat{r}}_{i}$ (all variants)	0.93	0.02	0.98	91%	10^−4^ [-0.05;0.05]
Experiment 1	${\hat{N}}_{e}^{h}$ (all dates)	0.86	28	0.91	90%	0.18 [-0.42; 1.48]
Experiment 2	${\hat{r}}_{i}$ (all variants)	0.92	0.06	0.94	89%	8.10^−4^ [-0.07;0.07]
Experiment 2	${\hat{N}}_{e}^{h}$ (all dates)	0.85	20	0.87	87%	0.03 [-0.53; 1.17]

^a^ Experiment 1: 750 simulated datasets with 5 variants under a wide range of selection and genetic drift regimes. Experiment 2: 350 simulated datasets with an additional and undetected sixth variant.

^b^ For each parameter, the determination coefficient *R*^2^, the slope and the intercept of the best linear model fit between predicted and true values are given, together with the percentage of true parameter values included in the 90% confidence interval and the mean relative bias and its 95% confidence interval.

In numerical experiment 2, we assessed the sensitivity of the estimation method to the presence of a sixth undetected virus variant (see S2 Text). This additional variant was neutral and initially present in the inoculum at a frequency of 3%. It affected virus population dynamics in all 48 host plants of the dataset, because we retained only Wright-Fisher simulations in which the frequency of this sixth variant ranged from 0.01 to 0.06 at 34 dpi. In the 350 simulated datasets, the mean frequency of the sixth variant at all sampling dates and in all plants was 0.07 (5% quantile = 0.01, median = 0.04, 95% quantile = 0.24). However, this variant was considered to be undetected by the HTS method. Thus, HTS analysis provided noisy estimates of the true frequencies of the five virus variants of interest: the mean relative difference between their true frequencies in the simulated population and their measured frequencies was 0.08 (5% quantile = 0.01, median = 0.05, 95% quantile = 0.29). Moreover, inference was performed assuming, as in numerical experiment 1, that the inoculum was an equimolar mixture of the five variants of interest. Despite this detection bias, the estimates of both $N_{e}^{h}\left(t_{d}\right)$ and *r*_*i*_ remained consistent (Table 2). The systematic presence of an undetected virus variant at a mean frequency of 0.07 only slightly affected the performance of the estimators (mean relative bias confidence intervals systematically included zero, the *R*^2^ of the best-fit lines remained unchanged, 90% of confidence intervals remained highly precise).

In a nutshell, from these numerical simulations with known parameter values, we can conclude that the proposed inference method provides accurate estimates of the intrinsic rates of increase *r*_*i*_ of each variant *i*, and, thus, of their selection coefficient, together with the dynamics of effective population size $N_{e}^{h}\left(t_{d}\right)$ during the time course of the experiment.

### Estimation of effective population sizes and variant fitness in the 15 plant genotypes

**Fig 5:**
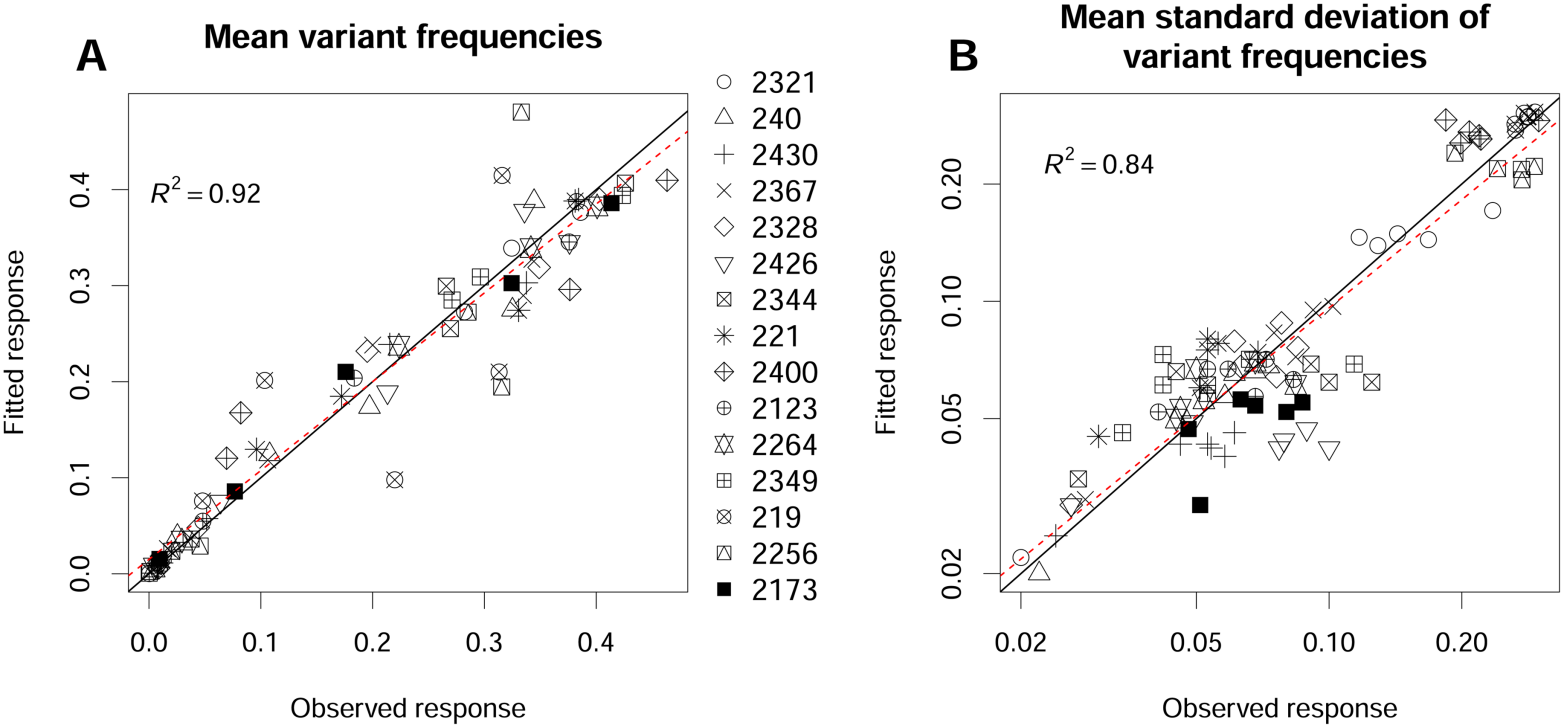
Goodness-of-fit of the Wright-Fisher model $M_{4}$ with the data of the biological experiments. (A) Correlation between the observed mean frequencies of the five virus variants (averaged over all virus populations and sampling times ($mean[f_{i}^{•}\left(•\right)]$) and their fitted values (*n* = 75). (B) Correlation between the logarithm of the observed mean (averaged over the variants) standard deviation of variant frequencies (between virus populations) at each sampling date *t*_*d*_ ($1/n_{var}\sum_{i=1}^{n_{var}}\sigma \left[f_{i}^{•}\left(t_{d}\right)\right]$) and their fitted values (*n* = 87). In both panels, the black line is the first bisector and the red dashed line is the best-fitting linear model.

We estimated the *N*_*e*_(*t*) and *r*_*i*_ of the PVY populations in each DH line with a Wright-Fisher model including selection and genetic drift. By contrast to the numerical experiments, the evolutionary parameters underlying the true dynamics of virus populations in their hosts were unknown. The Wright-Fisher model fitted the data very satisfactorily (Fig 5). The best-fit line between observed and fitted mean variant frequencies (averaged over all virus populations and sampling times) was very close to the first bisector (Fig 5A; slope = 0.92, intercept = 0.01, *R*^2^ = 0.92). This was also the case for the variability of variant frequencies between virus populations at each sampling date *t*_*d*_ (Fig 5B; slope = 0.92, intercept = -0.09, *R*^2^ = 0.84). A Wright-Fisher model including selection and genetic drift accurately described the mean evolutionary dynamics of a virus population and the variability of these dynamics between hosts. Due to an identifiability issue (we observed the relative frequencies of variants rather than variant densities), we had to fix the number of generations per day *γ*. We set this number to 1, a value close to that reported by Khelifa *et al.* [40]. Different *γ* values would change *r*_*i*_ and *N*_*e*_(*t*) estimates to $r_{i}^{1/\gamma }$ and *γN*_*e*_(*t*), but would have no effect on their ranking.

**Fig 6:**
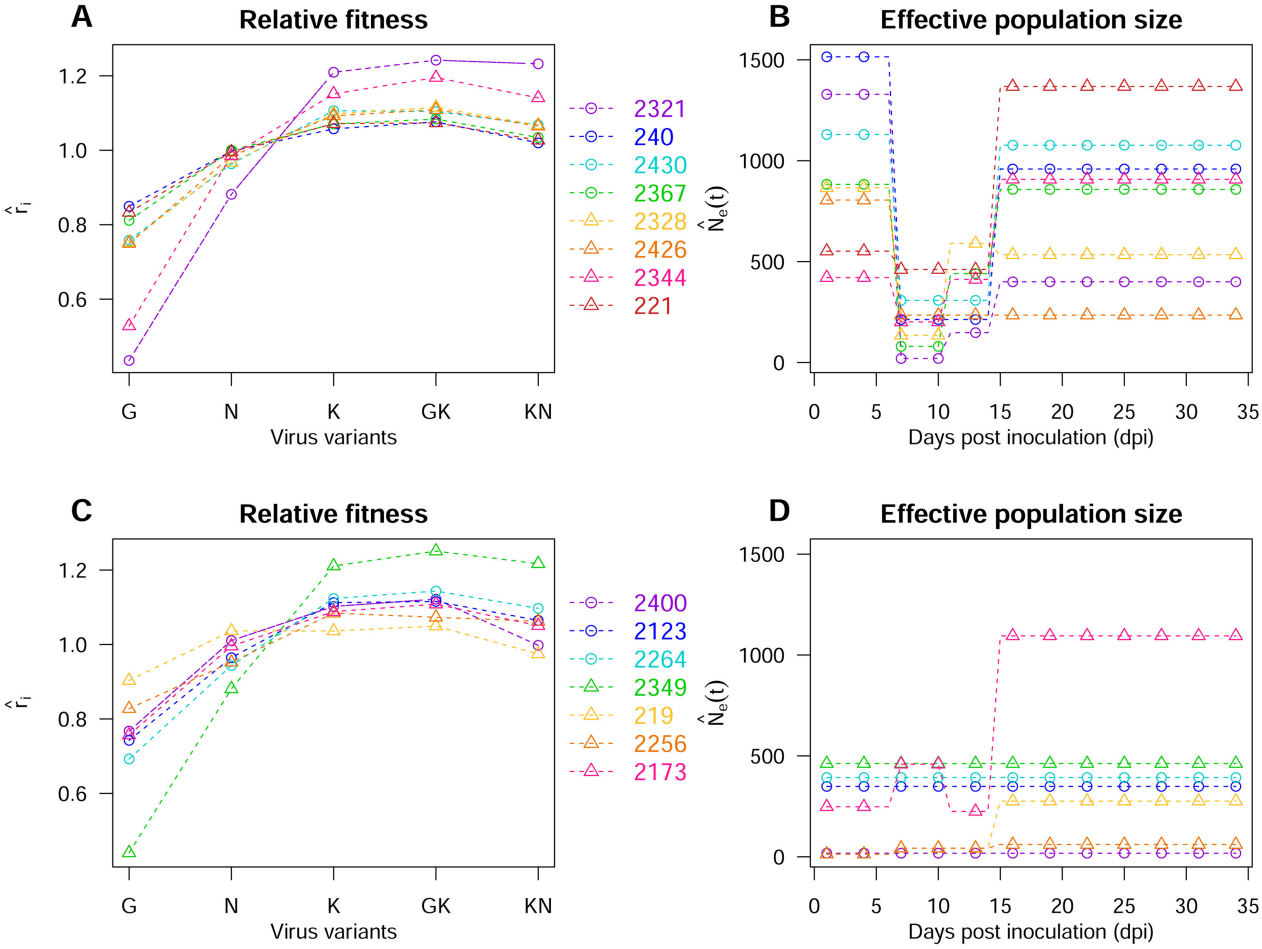
Fitness of virus variants and effective population size estimates for the 15 plant genotypes. (A) Estimates of intrinsic rates of increase ${\hat{r}}_{i}$ for each variant *i* for the DH lines 2321, 240, 2430, 2367, 2328, 2426, 2344 and 221. (B) Estimates of effective population size ${\hat{N}}_{e}\left(t\right)$ during the time course of the experiment for the DH lines listed in (A) and the model best supported by the data. (C) As for (A) for DH lines 2400, 2123, 2264, 2349, 219, 2256 and 2173. (D) As for (B) for the DH lines listed in (C).

Relative fitness values (*r*_*i*_) ranged from 0.43 to 1.25 (corresponding to |*s*|: 5% quantile = 0.004, mean = 0.12, 95% quantile = 0.27) and were associated with narrow 90% confidence intervals (S3 Table). The fitness ranks of the PVY variants were very similar in most DH lines (Fig 6A and 6C). Variant G was the weakest in all DH lines, followed by variant N in 13 DH lines. Variant GK was the fittest variant in 13 DH lines, with variant K the fittest variant in the remaining two lines (DH lines 2256 and 2430). Overall, variants K and GK were the two fittest variants in 12 DH lines; variants GK and KN were the two fittest in DH lines 2349 and 2321, and variants N and GK the two fittest in DH line 219. The fitness difference between the weakest and the fittest variants ranged from 0.14 for DH line 219 to 0.81 for DH line 2349.

We further estimated the dynamics of effective population size over the time course of the experiment, as modeled by a piecewise function *N*_*e*_(*t*), using a model selection procedure. Four models with one to four parameters were considered. The most general model $M_{4}$ distinguished four successive effective population sizes (one in the inoculated organ and three during systemic infection). $M_{4}$ was the model best supported by the data for five DH lines (2173, 2321, 2328, 2344 and 2367). Model $M_{3}$ distinguished three successive effective population sizes (one in the inoculated organ and two during systemic infection). It was best supported by the data for five DH lines (219, 221, 2256, 240 and 2430). Model $M_{2}$, which distinguished two successive effective population sizes (one in the inoculated organ and one during systemic infection), was selected for a single DH line (2426). Finally, with $M_{1}$, the effective population size of the virus population remained constant. This model was selected in the four remaining DH lines (2123, 2264, 2349 and 2400). The corresponding posterior probabilities of each model are shown in S4 Table, together with effective population size estimates and 90% credibility intervals.

At the first sampling date, considerable variability was observed (Fig 6B and 6D), with effective population sizes ranging from 13 for DH lines 219 and 2256 to 1515 for DH line 240. This was not surprising, given that we chose the DH lines on the basis of the density of primary infection foci in inoculated organs [34] (S1 Fig). A much narrower range of effective population sizes, from 18 to 462, was observed across all plant genotypes at 10 dpi, the first date on which systemic infection was observed. From 6 to 10 dpi, effective population sizes decreased in eight DH lines (Fig 6B), remained approximately constant in six DH lines (Fig 6D) and increased slightly in a single plant genotype (DH line 2173, Fig 6D). Later on, from 10 to 34 dpi, effective population size increased in eight DH lines (mostly DH lines displaying a bottleneck from 6 to 10 dpi, Fig 6B) and remained approximately constant in the others (mostly in DH lines with lower, *i.e.* < 500, effective population sizes in the inoculated organ, Fig 6D).

### Heritability of the intensities of selection and genetic drift exerted by plants on virus populations

By creating two dataset replicates of 24 randomly chosen plants for each DH line, we estimated the heritability of two plant traits corresponding to the evolutionary forces exerted by the plant on virus populations: selection and genetic drift. These forces were estimated by (i) intrinsic rates of increase in viral variants and (ii) effective population sizes for PVY. With 24 plants in each dataset, we used the function *N*_*e*_(*t*) of model $M_{2}$ with two parameters. In this approach, we used the contrasting behavior of PVY populations, which were fixed and identical at the time of inoculation in all plants, on different pepper genotypes to characterize the phenotype of each host. Very high heritability estimates were obtained for the intrinsic rates of increase (mean heritability over the five variant estimates: *h*^2^ = 0.94). Somewhat lower, but nevertheless substantial heritability estimates were obtained for effective population size in the inoculated organ (mean heritability, *h*^2^ = 0.64) and for effective population size during systemic infection (mean heritability, *h*^2^ = 0.63). The details of the calculation are provided in S3 Text.

## Discussion

Advances in sequencing technologies are revolutionizing the study of microbial evolution [13]. To our knowledge, this study is, for example, the first to suggest such strong variability in the selection and genetic drift regimes experienced by plant viruses in closely related host genotypes (Fig 2). This new type of data paves the way for the estimation of population genetics parameters influencing the fate of pathogen variants of special interest in medicine and agriculture (e.g. variants resistant to pesticides and drugs [44] or, as in this study, variants adapted to host resistance genes). However, estimation methods encompassing the whole range of variation of these parameters are still lacking.

### A method for estimating genetic drift and selection from microbial experimental evolution

We present here a method for the estimation of selection and genetic drift in a haploid and asexual organism, as modeled by a Wright-Fisher process. As for any model-based approach, the population of interest must not be too far from an ideal Wright-Fisher population with suitable parameters [10]. The estimation method did not require neutral markers. It was validated for small effective population sizes (*N*_*e*_ ≪ 100) and a wide range of both positive and negative selection coefficients (weak (|*s*| ≃ 0.01) or strong (|*s*| ≃ 0.15) selection), using simulated datasets. Recent reviews [23, 32] have highlighted the small number of methods available for the inference of selection and genetic drift over the whole range of variation, particularly in the case of small effective population sizes (*N*_*e*_ ≪ 1000) and strong selection coefficients (|*s*| ≃ 0.1). Indeed, these conditions do not fulfill the hypotheses underlying most approximations of the Wright-Fisher model. The classical approximation, with a standard diffusion process, requires both selection and genetic drift to be weak [23]. Approximations based on Gaussian diffusion require the stochastic effects of genetic drift to decrease more rapidly than the effects of selection [23]. The work of Foll *et al.* [11, 32] constituted a major step forward, but their method requires a large proportion of the genetic markers studied to be neutral. This assumption is not valid for many pathogens with small genomes, such as viruses. For example, only 22.7% of 66 randomly chosen mutations in the genome of *Tobacco etch virus* (TEV, genus *Potyvirus*), a plant RNA virus, were found to be consistent with neutrality [45]. As the statistical power to detect departure from neutrality is limited, the true proportion of neutral mutations is probably much lower. Similar results have been obtained for bacteria (e.g. [46]).

The estimation method proposed does not require neutral markers, an appealing feature for studying pathogens with small genomes. Lacerda and Seoighe [47] recently developed another method that does not require neutral markers. Their method provided satisfactory estimates of both *N*_*e*_ and *s* (estimated at a single locus) for a relatively small effective population size of 1000 individuals and values of *s* up to 0.5. They did not test the performance of their method for *N*_*e*_ ≪ 1000. By comparison, the method developed here was effective for much lower *N*_*e*_ values, in the range of a few tens of individuals, and for inferring the time course of *N*_*e*_ over a few tens of generations. However, although the range of selection coefficients *s* included cases of strong selection (|*s*| ≃ 0.1, as defined by Malaspinas [23]), none of the simulation experiments included values as high as 0.5. It may be possible to infer such high selection coefficients with the estimation method proposed, provided that the first generations are sampled more densely, typically every day after inoculation in our set-up. Lacerda and Seoighe [47], for example, used samples taken at each generation, for 20 generations. This makes it possible to record the trajectories of variant frequencies before variant loss or fixation.

The use of the proposed estimation method requires observation of the evolution of isolated populations derived from the same parental population, each population being sampled only once. This design is particularly suitable for studying within-host microbial evolution when several genetically-identical hosts (48 plants for each pepper genotype in our case study) can easily be included in the experiment. With this experimental design, we observed a set of variant frequencies at several time points, in independent hosts. This set contained footprints of selection and genetic drift. In the method developed, selection is evaluated from the mean trajectories of variant frequencies. Genetic drift is evaluated at several time points, by assessing differences in variant frequencies between the replicated populations during the time-course of the experiment. Even for populations with small effective sizes, for which genetic drift and selection have confounding effects on the fate of variants (Fig 2), a moderate number of replicates contains sufficient information to disentangle the two mechanisms. Here, we estimated four selection coefficients and four effective population sizes (*i.e.* 8 parameters) with 48 samples (6 sampling dates × 8 replicates).

The proposed estimation method could be improved further. It explicitly accounts for the technical sampling noise resulting from the assessment of variant frequencies from finite counts of virus sequences. However, HTS also introduces sequencing errors, albeit at a low rate of about 1 substitution per 400 bases for MiSeq technology [48], which were not explicitly accounted for in our framework. Several models have been proposed for separating true genetic variation from technical artifacts [48], and these models could be integrated into the method through a hierarchical Bayesian modeling framework [49], for example. Finally, the method could be extended to take mutation and recombination into account, particularly for experiments over longer periods, in which new variants might appear and displace those currently most abundant. In our short-term experiment, we have already observed *de novo* substitutions in a few plants (removed plant samples, see S1 Text). The inclusion of recombination is not relevant for our case study, as the nucleotide positions differentiating the variants are located only a few codons apart. Recombination can thus be ignored in this study [50], particularly given the small number of generations considered [32].

### Plant genotypes modulate genetic drift and selection within virus populations

On the host side, our experiment involved 15 DH lines of pepper, all carrying the major resistance gene *pvr2^3^*, but differing in terms of their genetic backgrounds [12]. These DH lines were derived from the *F*_1_ hybrid between two pepper lines, Perennial and Yolo Wonder. Consequently, on average, any pair of DH lines have 50 percent of alleles in common for markers differentiating between Perennial and Yolo Wonder. This is the first study, to our knowledge, to show such a high level of diversity in selection and genetic drift regimes experienced by virus populations from the same viral inoculum in closely related host genotypes (Fig 2, S2 and S3 Figs). On the pathogen side, we used five virus variants: the G and N variants displayed weaker adaptation to *pvr2^3^* than the K, GK and KN variants. The ranking of the selection coefficients of the five variants was mostly identical in the 15 plant genotypes. We were therefore unable to identify any host genotype, among those tested, able to counterselect against the virus variants best adapted to *pvr2^3^*. This may be due to (i) the strong selective effect exerted by the major-effect resistance gene *pvr2^3^*, which is present in all the DH lines studied here and probably exceeds the additional selective effect of the plant genetic background and/or (ii) the close genetic relatedness of the DH lines analyzed. Other genetic resources for pepper should be explored, to identify genotypes capable of counterselecting against the K, GK and KN variants, which were the fittest in our study. The best candidates for this would be pepper genotypes carrying *pvr2* resistance alleles other than *pvr2^3^*, with a different specificity in the face of PVY diversity [51], or pepper genotypes devoid of resistance alleles at the *pvr2* locus, as shown by Quenouille *et al.* [12]. Combinations of plant genotypes exerting opposite selective pressures on pathogen populations are particularly interesting for the sustainable management of plant resistance at landscape level, and can be implemented in cultivar rotations, mixtures or mosaics [52]. However, in our study, the difference in fitness between the weakest and fittest variants differed between host genotypes. The dynamics of selection for the fittest variants were under plant genetic control and could therefore be modulated by the choice of plant genotypes grown. For example, growing the pepper DH lines with the smallest differential selection between the five PVY variants would be particularly useful for delaying PVY adaptation in *pvr2^3^*-carrying plants, in which a two-step mutational trajectory may be required [12]. Indeed, the G and N variants are most likely to appear initially, because they require transitions, whereas the K variant requires a transversion, and transitions are more frequent than transversions [53]. However, an additional substitution, in a second step, is required to confer a sufficient level of fitness for the emergence of GK and KN variants. These mutational trajectories were observed in PVY adaptation to the Perennial pepper genotype, the resistant parent of all the DH lines studied here [12].

We also inferred the time course of the genetic drift experienced by the viruses in the 15 host environments during the experiment. Genetic drift intensities were highly variable with time and between plant genotypes, revealing an unprecedented level of variability between closely related host genotypes. Our estimates of *N*_*e*_(*t*) ranged from 18 to 462 just after the colonization of apical leaves at 10 dpi, and from 13 to 1515 in the inoculated leaves four days previously (at 6 dpi). Eight of the 15 DH lines displayed a high *N*_*e*_ in the inoculated leaves at 6 dpi (from 421 to 1515), a decrease at 10 dpi (*N*_*e*_ (10 dpi) values of 1.5 to 83.5% of the value at 6 dpi) and a subsequent increase (Fig 6B). This pattern suggests a founder effect, in which a new PVY population in apical leaves is set up by a few members of the original population in the inoculated leaf. In the remaining seven DH lines, the *N*_*e*_ of the inoculated leaves at 6 dpi was much lower (from 13 to 462), and *N*_*e*_ values often remained low in the apical leaves (Fig 6D). However, an increase in *N*_*e*_ was observed in DH lines 219 and 2173, after 14 dpi. This result sheds new light on the importance of the within-host bottlenecks experienced by virus populations, as discussed in a recent article by Zwart *et al.* [54], who reported that the *N*_*e*_ of TEV in the first systemically infected leaf of tobacco plants was determined largely by inoculum viral load. They then hypothesized that genetic drift occurred mostly during the inoculation process. Previous estimations of *N*_*e*_ for viruses did not focus on *N*_*e*_ dynamics at the whole-plant level as in this study. Instead, they considered the multiplicity of infection (MOI) during cell-to-cell movement or *N*_*e*_ during the colonization of apical leaves (for a comprehensive review, see Gutiérrez *et al.* [4]). Direct comparisons with these studies are, therefore, not appropriate. Gutiérrez *et al.* [55] recently showed that *Turnip mosaic virus* (genus *Potyvirus*) infections are characterized by a very low MOI (≃ 1) when cells are infected with virus particles moving in the plant vasculature, and a much higher MOI (≃ 30) during subsequent cell-to-cell movement in the mesophyll. The general picture that emerges when we consider both these MOI patterns and plant growth dynamics is consistent with our observations. Indeed, the lowest *N*_*e*_ values were observed at 10 dpi, corresponding to the onset of systemic infection, when plants were small and consisted essentially of a few infected leaves. *N*_*e*_ tends often to increase with time, because (i) increasing numbers of leaves are infected and behave as virus sources as the plant grows and (ii) leaf areas increase, probably increasing the relative proportion of cell-to-cell, as opposed to long-distance, virus movement.

One of the key results of this study is the finding that the effective population size of PVY is a heritable plant trait. The high heritability estimated for *N*_*e*_ (partially due to the use of a DH progeny of pepper genotypes) indicates that plant resistance could potentially be improved through breeding programs. Indeed, our findings pave the way for the breeding of plant cultivars exposing viruses to greater genetic drift. This would provide a twofold benefit against viruses. First, in asexual populations, genetic drift favors the accumulation of deleterious mutations, decreasing viral fitness (Muller’s ratchet) [56]. Second, genetic drift decreases the fixation probability of beneficial mutations, such as those responsible for overcoming plant resistance genes [57]. Breeding for greater genetic drift in virus populations would thus constitute a novel approach to increasing the durability of resistance to plant viruses in agricultural landscapes [52, 58, 59]. Another key result is the finding that the Wright-Fisher model accurately captures the major processes driving the within-host dynamics of a set of virus variants (Fig 5), despite being much simpler than the underlying mechanisms involved in the infection of highly structured hosts. Over longer periods, mutation and recombination increase in importance and this can easily be encompassed in the Wright-Fisher model [60]. This model can thus serve as a valuable cornerstone for linking the within- and between-host scales of disease dynamics and studying, for example, how breeding for greater genetic drift can delay the emergence of a new pathogen variant.

## Supporting information

S1 Text Sequence analyses to detect PVY mutations. (PDF)

S2 Text Numerical experiments. (PDF)

S3 Text Heritability of the intensity of selection and genetic drift exerted by plants on virus populations. (PDF)

S1 Fig Resistance-breakdown (RB) frequency, viral accumulation and mean number of primary infection foci for the 15 DH lines studied. Pepper genotypes are represented as points, with their nomenclature (DH line number) given above each point. We estimated the mean number of primary infection foci for the 15 DH lines with the *Potato virus Y* (PVY, genus *Potyvirus*) variant K, carrying a green fluorescent marker (green fluorescent protein, GFP) [34]. The resistance-breakdown (RB) frequency and the relative viral accumulation were estimated by Quenouille *et al.* [12]. The RB frequency corresponds to the percentage of infected plants when inoculated with an avirulent variant regarding the allele of resistance *pvr2^3^*, carried by all DH lines. The relative viral accumulation, or relative viral concentration, was measured by double antibody sandwich enzyme-linked immunosorbent assay (DAS-ELISA).
(PDF)

S2 Fig Five datasets obtained by high-throughput sequencing in the biological experiment. Each line of bar plots represents the dynamics of virus variants in a single DH line over time: (A) 221, (B) 2123, (C) 2173, (D) 2256 and (E) 2264. Within each bar plot, the frequencies of the five variants (see top of the figure for the color code) in each infected plant sample are represented by single bars (labeled from 1 to 48). The missing bars correspond to plant samples for which no viruses were detected. The last bar indicates the mean viral composition in the infected plants. Each individual bar plot corresponds to a single sampling date, indicated at the top of each column of barplots.
(PDF)

S3 Fig Five datasets obtained by high-throughput sequencing in the biological experiment. Each line of bar plots represents the dynamics of virus variants in a single DH line over time: (F) 2328, (G) 2349, (H) 2367, (I) 2400 and (J) 2426. Within each bar plot, the frequencies of the five variants (see top of the figure for the color code) in each infected plant sample are represented by single bars (labeled from 1 to 48). The missing bars correspond to plant samples for which no viruses were detected. The last bar indicates the mean viral composition in the infected plants. Each individual bar plot corresponds to a single sampling date, indicated at the top of each column of barplots.
(PDF)

S4 FigVariability of the selection and genetic drift regimes obtained among the simulated datasets in numerical experiment 1.In the diagonal, each histogram represents the distribution of input parameters *r*_1_ (intrinsic rate of increase of variant 1), $N_{e}^{IO}$ (effective population size in the inoculated organ) and $N_{e}^{S_{1}}$ (effective population size at the onset of the systemic infection) used to simulate the 750 datasets. Off-diagonal scatter plots are two by two combinations of parameters.(PDF)

S1 Table Tag sequences used to distinguish each plant sample after pooling and MiSeq Illumina high-throughput sequencing. The forward (Fwd.) primer sequence was the same for all amplifications and was bound to the sequence tag, just after it. Its binding site corresponds to positions 5971 to 5990 of PVY isolate SON41p (accession number AJ439544). The binding site of the reverse (Rev.) primer sequence corresponds to positions 6095 to 6114 of PVY isolate SON41p. RT-PCR amplifications were done according to the following profile: 1h at 42°C, 10 min at 95°C, 35 times the following sequence (45s at 95°C, 30s at 50°C and 20s at 72°C) and finally 10 min at 72°C.
(PDF)

S2 Table Number of sequences and composition of the virus populations in each sample of the biological experiment. In all, 708 samples were analyzed: 15 doubled-haploid (DH) lines of pepper × 6 sampling dates (dpi: days post-inoculation) × 8 plants per date, except for virus-negative samples, and 4 samples for the initial inoculum. Columns indicate (i) the name of each DH line, (ii) the sampling date in dpi, (iii) the number of the sequence tag used (see S1 Table), (iv) the plant number (as in Fig 2, S2 and S3 Figs), (v) the infection status of each sample (0: not infected / 1: infected), (vi) the total number of cleaned sequences of variants G, N, K, GK and KN assigned to each sample after filtering, (vii-xi) the number of sequences for each inoculated viral variant (G, N, K, GK and KN), and (xii-xiv) the number of sequences of the three additional variants SON41p, GN and GKN, based on the three codon positions of interest in the VPg cistron.
(XLSX)

S3 TableEstimations of the relative intrinsic rates of increase of the virus variants for the 15 plant genotypes.Virus variants are indexed as follows: (i) *r*_1_ virus variant G, (ii) *r*_2_ virus variant N, (iii) *r*_3_ virus variant K, (iv) *r*_4_ virus variant GK, (v) *r*_5_ virus variant KN. The 90% confidence intervals are calculated as $\hat{r_{i}}\pm 1.645.\hat{\sigma_{i}}$.(PDF)

S4 TableModel selection and estimations of the effective population sizes for the 15 plant genotypes.The posterior probabilities of the four models considered for the piecewise function describing the temporal variation of the effective population sizes during the time course of the experiment (models $M_{1}$, $M_{2}$, $M_{3}$ and $M_{4}$) are first indicated. The bold value corresponds to the model that is best supported by the data. The next columns indicate the estimation of the effective population sizes of the model selected and the extent of the 90% credibility intervals.(PDF)
