# Supplementary material for: Estimating virus effective population size and selection without neutral markers
Source: PLoS Pathog. 2017 Nov 20;13(11):e1006702. doi: 10.1371/journal.ppat.1006702 (PMC5720836; doi:10.1371/journal.ppat.1006702)
Supplement: S1 Text — (PDF) [file ppat.1006702.s001.pdf]

Supplementary Information – Text S1: Sequence analyses to detect PVY mutations

Viruses being characterized by high mutation rates, we conducted a sequence analysis to detect potential mutations in virus populations representing the common inoculum and the infected plants. In all, 677 samples were analyzed, corresponding to all infected plants from the 15 doubled-haploid (DH) lines of pepper, and 4 samples representing replicates of the initial inoculum. This analysis is important because the presence of mutants could affect the dynamics of virus populations and the intensities of the evolutionary forces at stake. We analyzed each nucleotide position in all sequences, starting from the end of the forward primer until the beginning of the reverse primer, i.e. 99 nucleotide positions per sequence. First, we focused on the three single-nucleotide polymorphisms (SNPs) located at codon positions 101, 115 and 119 of the VPg cistron which distinguish the five variants mixed to make the inoculum, i.e. variants G, N, K, GK and KN (see Fig. 1A in main text). For each sequence, we determined the corresponding variant among the eight possible (2^3) at the three nucleotide positions of interest. By doing so, we could estimate the frequencies of the five variants included in the inoculum and of the three possible variants carrying other SNP combinations at the three nucleotide positions of interest (i.e. the wild-type variant SON41p and variants GN and GKN). Then, for each of the 677 PVY populations, we determined the relative frequencies of these eight PVY variants. The additional three possible variants SON41p, GN and GKN could have appeared by mutation or recombination, either *in vivo* or *in vitro*, and should thus be surveyed. In a second step, we calculated the frequencies of all remaining nucleotide substitutions in each virus population by comparison with the sequence of the SON41p reference clone (equivalent to comparison with sequences of the G, N, K, GK and KN clones).

Sequence counts of the eight variants corresponding to the SNPs present in the initial inoculum We assigned each sequence to one of the eight potential PVY variants defined by the three SNPs of interest (variants G, N, K, GK, KN, SON41p, GN or GKN, see Fig. 1A in main text). Sequence counts are available in Table S2.

The sum of the frequencies of the three variants SON41p, GN and GKN that had not been included into the inoculum remained below 5% in all virus populations analyzed (Table 1). Additionally, 93.35% of samples showed a sum of frequencies below 2%, and 99.41% were below 3%. In the inoculum, the sum of the mean frequencies of these three variants was 2.32% (SON41p: 1.13%, GN: 0.50%, GKN: 0.69%), while it was 1.03% in plant samples (SON41p:

0.14%, GN: 0.11%, GKN: 0.78%). Given the low frequencies recorded, we cannot tell if those sequences indeed correspond to variants present in the virus population, or if they are artifacts due to errors during RT-PCR or sequencing. Negroni and Buc reported estimates of the recombination frequency by reverse transcriptases between $4 \cdot 10^{-5}$ and $2.4 \cdot 10^{-4}$ per nucleotide [6]. Our experimental framework involved one reverse transcription (RT) step and a maximum distance of 55 nucleotides between SNPs (corresponding to the G and N substitutions), yielding a recombination probability of 0.2 to 1.3%. We recorded 76.66 to 99.41% of samples with frequencies of variant SON41p below 0.2 to 1.3%, respectively. For the same variant frequencies (0.2% and 1.3%), the sample frequencies reached 79.32 to 99.85% for variant GN and 13.15 to 84.64% for variant GKN. Additionally, Potapov and Ong [7] estimated a polymerase chain reaction (PCR)-mediated recombination rate by *Taq* polymerase of $1.1 \cdot 10^{-4}$ per nucleotide and per doubling. For one doubling cycle, and considering again the most distant SNPs (55 nucleotides), the probability for one recombination event would be of 0.6%. We performed 47 PCR cycles, 35 to amplify the raw PCR products plus 12 to introduce the indices that identify each sequenced virus population. The expected recombination probability would be of 24.64%. Hence there is a high probability of artifactual presence of recombinants. This figure corresponds to the recombination probability between two types of DNA molecules present in equal proportion, which is not the case in our experiment comprising five different kinds of DNA molecules with highly variable frequencies. Moreover, recombinant DNA molecules generated *in vitro* may themselves recombine with other DNA molecules in the following PCR cycles. Consequently, although we expect high probabilities of *in vitro* recombination, these probabilities should be lower than the 24.64% estimated. In addition, given that these three variants could also have been generated by mutation of some of the five initial variants, and given the mutation error rate of RT, PCR or Illumina sequencing (a maximum estimated value of 0.86%; see below), it is not possible to distinguish these three PVY variants from the error background of laboratory enzymes. Additional arguments come from the comparison between the frequencies of the SON41p, GN and GKN variants in the inoculum and in infected plants. Concerning the inoculum population, there was no opportunity for *in vivo* recombination between the G, N, K, GK and KN variants, since these five variants were multiplied separately in different plants before being mixed to make the inoculum. Consequently, the SON41p, GN and GKN variants detected in the inoculum have been generated either by *in vitro* recombination or by mutation (*in vivo* or *in vitro*). In contrast, *in vivo* recombination may have been involved in the generation of these variants in the infected plants of the experiment, in addition to the previous two mechanisms. The fact that the frequency of the three putative recombinants is

lower in infected plants than in the inoculum indicates that *in vivo* recombination was negligible. Furthermore, even if some of these recombinants had been truly generated *in vivo*, their decrease in frequency between the inoculum and infected plants indicates that those variants did not play a substantial role in virus dynamics.

Table 1: **Counts of the sum of the frequencies of the three variants SON41p, GN and GKN in virus populations. Only intervals where at least one case was observed are reported.**

**Interval (%)**	]0-1]	]1-2]	]2-3]	]3-4]	]4-5]	**Total**
**Counts**	352	280	41	3	1	677

Frequencies of *de novo* nucleotide substitutions The complementary analysis consisted in looking at substitutions at all remaining 96 nucleotide positions, and at the two possible remaining substitutions at the three SNP positions of interest, i.e. those that were absent in the inoculum (first part). We focused on the sum of the frequencies of potential *de novo* substitutions at each nucleotide position, in plant samples or in the inoculum (Table 2). In infected plants, 5.15% (3452/67023) of nucleotide positions did not show any *de novo* substitution (i.e. frequency of 0%). Additionally, 99.91% of nucleotide positions showed a frequency of *de novo* substitutions below 1%, and 99.99% showed a frequency below 5%.

Table 2: **Counts in various intervals of the sum of the frequencies of all potential *de novo* substitutions at each nucleotide position, in plant samples and in the inoculum (inoc.). Only intervals where at least one case was observed are reported.**

**Interval (%)**	0	]0-0.1]	]0.1-0.2]	]0.2-0.3]	]0.3-0.4]	]0.4-0.5]	]0.5-1]	]1-2]	]2-3]
**Counts in plants**	3452	14817	22413	15488	7212	2493	1091	39	6
**Counts in inoc.**	6	87	171	94	28	8	2	0	0
**Interval (%)**	]3-4]	]4-5]	]5-6]	]6-7]	]14-15]	]26-27]	]71-72]		**Total**
**Counts in plants**	2	3	2	2	1	1	1		67023
**Counts in inoc.**	0	0	0	0	0	0	0		396

Illumina MiSeq sequencing errors comprise mostly nucleotide mismatches and a much lower rate of insertions or deletions [9]. The mismatch error rate was estimated between 0.25 and 0.46% per nucleotide [4, 9]. Besides, the mismatch error rates of the RT and PCR steps should be added to the mismatch error rate of Illumina MiSeq. The error rate of *Avian myeloblastosis* virus reverse transcriptase (0.0027% per nucleotide [8]) appears negligible compared to those of Illumina MiSeq and of the *Taq* polymerase used for PCR (0.27 to $0.85 \cdot 10^{-4}$ error per base pair and per cycle) [3]. Over the 47 PCR cycles carried out in our experiments, this *Taq*

polymerase would yield 0.13 to 0.40% of chances of cumulating at least one error per base pair. Taking into account the maximum error rate of Illumina MiSeq sequencing (0.46%) and of PCR (0.40%), we expect a total maximum error rate of 0.86% per nucleotide in our experiment. The vast majority (99.87%) of the sum of frequencies of substitutions at each nucleotide position recorded fall below this estimated error rate. Hence, given this threshold, most substitutions recorded probably result from *in vitro* errors during RT-PCR or Illumina MiSeq sequencing rather than being real substitutions occurring during virus replication.

Globally, the sum of the frequencies of *de novo* substitutions per nucleotide position varied between 0 and 71.52% in plant samples, while they reached at most 0.63% in the inoculum. Hence, in some plants, mutations have appeared and largely spread in the virus population during infection. Nevertheless, those cases are rare as only seven observations show a sum of frequencies of substitutions at a nucleotide position above 5% (Table 2), and six observations with a frequency of a single substitution (not the sum) above 5%. Details about those six latter cases are reported in Table 3.

Interestingly, three out of the six cases showed the same substitution at codon position 120 (Table 3). Three amino-acid substitutions at this position (serine to cysteine, isoleucine or threonine) have already been reported as determining adaptation to the *pvr2*^{3}-mediated resistance in several studies [1, 2, 5] and unpublished data, but not the serine (S) to arginine (R) substitution observed here. It is therefore highly likely that the $S_{120}R$ substitution was also positively selected in the *pvr2*^{3} host environment. Let us also note that one *de novo* substitution was observed at one of the three SNP positions of interest, corresponding to codon position 115 but yielded a different amino acid (arginine) than the one characterizing variant K (lysine) or SON41p reference clone (threonine). Substitution to arginine at codon 115 was shown previously to confer PVY adaptation to the *pvr2*^{3} resistance [1]. Interestingly, among the DH lines concerned by the highest frequencies of *de novo* substitutions, lines 2256 and 2400 are part of the ones inducing strongest genetic drift on virus populations, and DH line 2264 induces intermediate genetic drift (Fig 5). Hence, it is possible that the new mutations observed benefited from population expansion after a bottleneck step during plant infection, and this founder effect could have allowed these mutations to reach rapidly high frequencies in the virus populations. As a precaution, those six plants were removed for the estimation of effective population sizes (N_e) and selection coefficients (s).

We kept all remaining plant samples, showing frequencies of substitution below 5% (Table 2) and only the five variants mixed in the inoculum (G, N, K, GK and KN) for N_e and s estimations. In a numerical experiment, we tested the impact of a sixth, not accounted for

Table 3: **The six most frequent *de novo* nucleotide substitutions observed (> 5%) in the PVY populations. Indicated are the doubled-haploid (DH) line concerned, day of sampling (days post-inoculation, dpi), plant number, codon position, codon and its reference (Ref.) from the original sequence, corresponding amino-acid (aa) and its reference, and mutation frequency in the sample.**

DH line	Dpi	Plant	Codon position	Codon (Ref.)	aa (Ref.)	%
2256	34	1	120	AGA (AGT)	R (S)	71.32
2400	27	3	95	GAG (GAT)	E (D)	25.94
2256	14	3	110	AAT (GAT)	N (D)	14.07
2400	27	2	115	AGG (ACG)	R (T)	6.20
2400	6	4	120	AGA (AGT)	R (S)	5.91
2264	10	6	120	AGA (AGT)	R (S)	5.08

variant starting at a frequency of 3% in the inoculum, being neutral, and still present at the last sampling date (Text S2). The mean frequency of this sixth variant at all sampling dates and in all plants was 7% (5% quantile = 1%, median = 4%, 95% quantile = 24%). Overall, the accuracy of N_e and s estimations was not significantly impacted by the presence of this sixth variant (see Table 2 in main text). Hence, we can also trust our biological estimations based on the frequencies of substitutions recorded and neglecting *de novo* substitutions or potential recombinants present on average at a frequency < 7%.

References

1. V. Ayme, S. Souche, C. Caranta, M. Jacquemond, J. Chadœuf, A. Palloix, and B. Moury. Different Mutations in the Genome-Linked Protein VPg of *Potato virus Y* Confer Virulence on the *pvr*2^3 Resistance in Pepper. *MPMI*, 19(5):557–563, 2006.

2. V. Ayme, J. Petit-Pierre, S. Souche, A. Palloix, and B. Moury. Molecular dissection of the potato virus Y VPg Virulence factor reveals complex adaptations to the *pvr2* resistance allelic series in pepper. *Journal of General Virology*, 88:1594–1601, 2007.

3. M. A. Bracho, A. Moya, and E. Barrio. Contribution of *Taq* polymerase-induced errors to the estimation of RNA virus diversity. *Journal of General Virology*, 79:2921–2928, 1998.

4. D. Laehnemann, A. Borkhardt, and A. C. McHardy. Denoising DNA deep sequencing data— high-throughput sequencing errors and their correction. *Briefings in Bioinformatics*, 17(1):154–179, 2016.

5. J. Montarry, J. Doumayrou, V. Simon, and B. Moury. Genetic background matters: a plant-virus gene-for-gene interaction is strongly influenced by genetic contexts. *Mol. Plant Pathol.*, 12(9):911–920, 2011.

6. M. Negroni and H. Buc. Mechanisms of retroviral recombination. *Annu. Rev. Genet.*, 35:275–302, 2001.

7. V. Potapov and J. L. Ong. Examining sources of error in PCR by single-molecule sequencing. *PLoS ONE*, 12(1):e0169774, 2017.

8. J. D. Roberts, B. D. Preston, L. A. Johnston, A. Soni, L. A. Loeb, and T. A. Kunkel. Fidelity of two retroviral reverse transcriptases during DNA-dependent DNA synthesis in vitro. *Molecular and Cell Biology*, 9(2):469–476, 1989.

9. M. G. Ross, C. Russ, M. Costello, A. Hollinger, N. J. Lennon, R. Hegarty, C. Nusbaum, and D. B. Baffe. Characterizing and measuring bias in sequence data. *Genome Biology*, 14:R51, 2013.
