# Supplementary material for: Estimating virus effective population size and selection without neutral markers
Source: PLoS Pathog. 2017 Nov 20;13(11):e1006702. doi: 10.1371/journal.ppat.1006702 (PMC5720836; doi:10.1371/journal.ppat.1006702)
Supplement: S2 Text — (PDF) [file ppat.1006702.s002.pdf]

Supplementary Information – Text S2: Numerical experiments

We performed several batches of simulations to assess the ability of the estimation method to infer effective population sizes and selection coefficients accurately. Two numerical experiments were performed.

Experiment 1. In experiment 1, we simulated datasets involving a population of 5 virus variants, initially in equimolar mixture and all detected by the high-throughput sequencing (HTS) method. A dataset was obtained with the following three steps: (1) random sampling of selection and genetic drift parameters, (2) generation of population demogenetic dynamics given these known parameters with a Wright-Fisher model including selection and genetic drift and (3) building of numerical datasets with a structure similar to our real-life experiment, by sampling the composition of several populations at various time-points.

Step 1: Random sampling of selection and genetic drift parameters. Parameters $\boldsymbol{\theta}_{true} = (\boldsymbol{r}_{true}, \boldsymbol{N}_{true})$ are independently drawn from dedicated distributions that encompass a large diversity of selection and genetic drift scenarios. The relative fitness of the 5 virus variants $\boldsymbol{r}_{true}$ is obtained by independently drawing 5 values in uniform distribution ($\sim$ Unif$[0.85, 1.15]$) and then dividing them by their mean in order to have mean$(\boldsymbol{r}_{true}) = 1$. The scenario of genetic drift is obtained as follows. Firstly, the effective population size in the inoculated organ (N_e^{IO}) is drawn in a log-uniform distribution ($\sim$ Log-unif$[10, 2000]$). This stage lasts 6 generations. Secondly, the effective population size at the onset of system infection ($N_e^{S_1}$) is drawn in a log-uniform distribution ($\sim$ Log-unif$[10, 2000]$). This stage lasts 5 generations. Then, 5 more effective population sizes are drawn, corresponding to systemic infection stages. In order to avoid unrealistic trajectories ([1]), we set that the ratio of population sizes between two consecutive stages could not exceed 10. In practice, we iteratively computed $\log10(N_e^{S_{i+1}}) = \max(\min(\log10(N_e^{S_i}) + \alpha_i, \log10(2000)), \log10(10))$, with α_i sampled uniformly between -1 and 1 and $1 \leq i \leq 5$. Each stage lasts 5 generations, except the last one lasting 3 generations. We thus obtained a vector $\boldsymbol{N}_{true}$ lasting 34 generations as follows:

$$\boldsymbol{N}_{true} = (\underbrace{N_e^{IO}, N_e^{IO}, \ldots,}_{\text{6 generations}} \underbrace{N_e^{S_1}, N_e^{S_1}, \ldots,}_{\text{5 generations}} \underbrace{N_e^{S_2}, \ldots}_{\text{5 generations}}, \ldots, \underbrace{N_e^{S_6}, \ldots}_{\text{3 generations}}) \quad (1)$$

Step 2: Generation of population demogenetic dynamics. We generated 48 independent Wright-Fisher simulations (using equations (2) and (3) of the main text) corresponding to the dynamics of populations of the 5 virus variants in 48 different plants of the same plant genotype, given $\boldsymbol{\theta}_{true} = (\boldsymbol{r}_{true}, \boldsymbol{N}_{true})$ and $\boldsymbol{\lambda}^{inoc} = (0.2, 0.2, 0.2, 0.2, 0.2)$.

Step 3: Building numerical datasets. We then carried out virtual observations for eight individual plants on each of the measurement dates corresponding to those used in the biological experiment, $\boldsymbol{T}^{obs}$ = (6, 10, 14, 20, 27, 34) days post-inoculation (dpi). We accounted for the HTS process, by sampling variant frequencies from multinomial distributions of size 3000 and with frequencies from Wright-Fisher simulations (in the same way as for step 2 of the ABC algorithm described in the main text). Importantly, in experiment 1, HTS analysis provides samples of the true frequencies of virus variants in the simulated Wright-Fisher populations. Finally, the dataset generated was accepted according to the following criteria, considered to be satisfied for all datasets from laboratory experiments (with the exception of one plant in 720). At each measurement date, at least two variants had to be present at a minimum frequency of 1% each in at least 50% of the populations. In addition, at least two variants had to be present in all populations at a minimum frequency of 1% each at the first measurement date (6 dpi). This criterion is hence largely permissive regarding the diversity of virus populations retained.

Step 4: Estimation of parameters. Using the three previous steps, we generated 750 datasets under as many selection and genetic drift regimes as defined by the corresponding 750 values of $\boldsymbol{\theta}_{true}$. In order to assess the ability of the estimation method to infer effective population sizes and selection coefficients accurately, we estimated for each dataset $\hat{\boldsymbol{\theta}} = (\hat{\boldsymbol{r}}, \hat{\boldsymbol{\eta}}_e)$ using the more general model $\mathfrak{M}_4$ (*i.e.* with $\boldsymbol{\eta}_e = \left(\eta_e^{IO}, \eta_e^{S_1}, \eta_e^{S_2}, \eta_e^{S_3}\right)$). We assessed the accuracy of the estimates by comparing directly the estimated values of intrinsic rates of increase $\hat{\boldsymbol{r}}$ with their true values $\boldsymbol{r}_{true}$. For the effective population sizes, we compared the true harmonic mean of effective population sizes assessed from $\boldsymbol{N}_{true}$ at each measurement date $\boldsymbol{T}^{obs}$ and the harmonic mean of effective population sizes assessed from the piecewise function $N_e(t)$ (equation (1) of the main text) parameterized by $\hat{\boldsymbol{\eta}}_e$.

Experiment 2. In experiment 2, we tested the sensitivity of the estimation method to the presence of a sixth undetected virus variant. This sixth variant was selectively neutral (its selection coefficient is null), present in the inoculum at a frequency of 3% and still present at

the last sampling date (34 dpi) in all plants analyzed at frequencies ranging from 1% to 6%. It impacts the dynamics of the 5 variants of interest in all plants but is not detected, meaning that variant frequencies measured by HTS are noisy with respect to their true values. In all, 350 simulated datasets were analyzed for this second test. The mean relative change between the true frequencies of the 5 variants of interest in the simulated population and their measured frequencies by HTS is 0.08 (5% quantile = 0.01, median = 0.05, 95% quantile = 0.29).

In practice, a dataset in experiment 2 was obtained with the previous three steps modified as follows. In step 1 (Random sampling of selection and genetic drift parameters), the only difference is to add a sixth relative fitness equal to 1 to the vector $\boldsymbol{r}_{true}$. In step 2 (Generation of population demogenetic dynamics), as many as necessary independent Wright-Fisher simulations given $\boldsymbol{\theta}_{true} = (\boldsymbol{r}_{true}, \boldsymbol{N}_{true})$ and $\boldsymbol{\lambda}^{inoc} = (0.194, 0.194, 0.194, 0.194, 0.194, 0.03)$ are performed until having 48 independent simulations where the sixth variant is still present at the last sampling date (34 dpi) at frequencies ranging from 1% to 6%. Then, the dynamics of the sixth variant is erased from the 48 independent simulations retained. Step 3 (Building numerical datasets) is the same as previously. However, due to the deletion of the dynamics of the sixth variant, the frequencies of the 5 virus variants of interest used to mimic HTS though multinomial sampling are no more the true frequencies of the variants in the virus population but noisy values. Step 4 is also the same as in experiment 1. In particular, the inference was performed assuming that the inoculum was an equimolar mixture of the 5 variants of interest.

References

1. Boitard S, Rodríguez W, Jay F, Mona S, Austerlitz F. Inferring Population Size History from Large Samples of Genome-Wide Molecular Data - An Approximate Bayesian Computation Approach. PLOS Genetics. 2016;e1005877.
