# Supplementary material for: Estimating virus effective population size and selection without neutral markers
Source: PLoS Pathog. 2017 Nov 20;13(11):e1006702. doi: 10.1371/journal.ppat.1006702 (PMC5720836; doi:10.1371/journal.ppat.1006702)
Supplement: S3 Text — (PDF) [file ppat.1006702.s003.pdf]

Supplementary Information – Text S3: Heritability of the intensity of selection and genetic drift exerted by plants on virus populations

Heritabilities were estimated by creating two dataset replicates of 24 randomly chosen plants for each DH line, each one including 4 plants at each sampling date. This random split was repeated 8 times in order to obtain 8 heritability estimates per variable of interest. Heritabilities were assessed for (i) the intrinsic rates of increase r_i of each virus variant i, (ii) the virus effective population size in the inoculated organ η_e^{IO} and (iii) the virus effective population size during systemic infection η_e^S. With 24 plants in each dataset, a simpler $N_e(t_g)$ piecewise function with only two parameters was used: $N_e(t) = \eta_e^{IO}$ when $t \in [1, 6]$) and (ii) $N_e(t) = \eta_e^S$ when $t \in [7, 34]$. The table below provides the mean heritabilities over the 8 repetitions of r_i (averaged over the 5 virus variants), η_e^{IO} and η_e^S. Additionally, the last column provides the range of variation of the heritabilities over the 8 repetitions.

Table 1: **Heritability estimations.**

**Variable**	**Mean heritability**	**Range of variation**
r_i	0.94	[0.76; 0.98]
η_e^{IO}	0.64	[0.36; 0.89]
η_e^S	0.63	[0.47; 0.85]
