# Supplementary figures and images for: Estimating virus effective population size and selection without neutral markers
Source: PLoS Pathog. 2017 Nov 20;13(11):e1006702. doi: 10.1371/journal.ppat.1006702 (PMC5720836; doi:10.1371/journal.ppat.1006702)

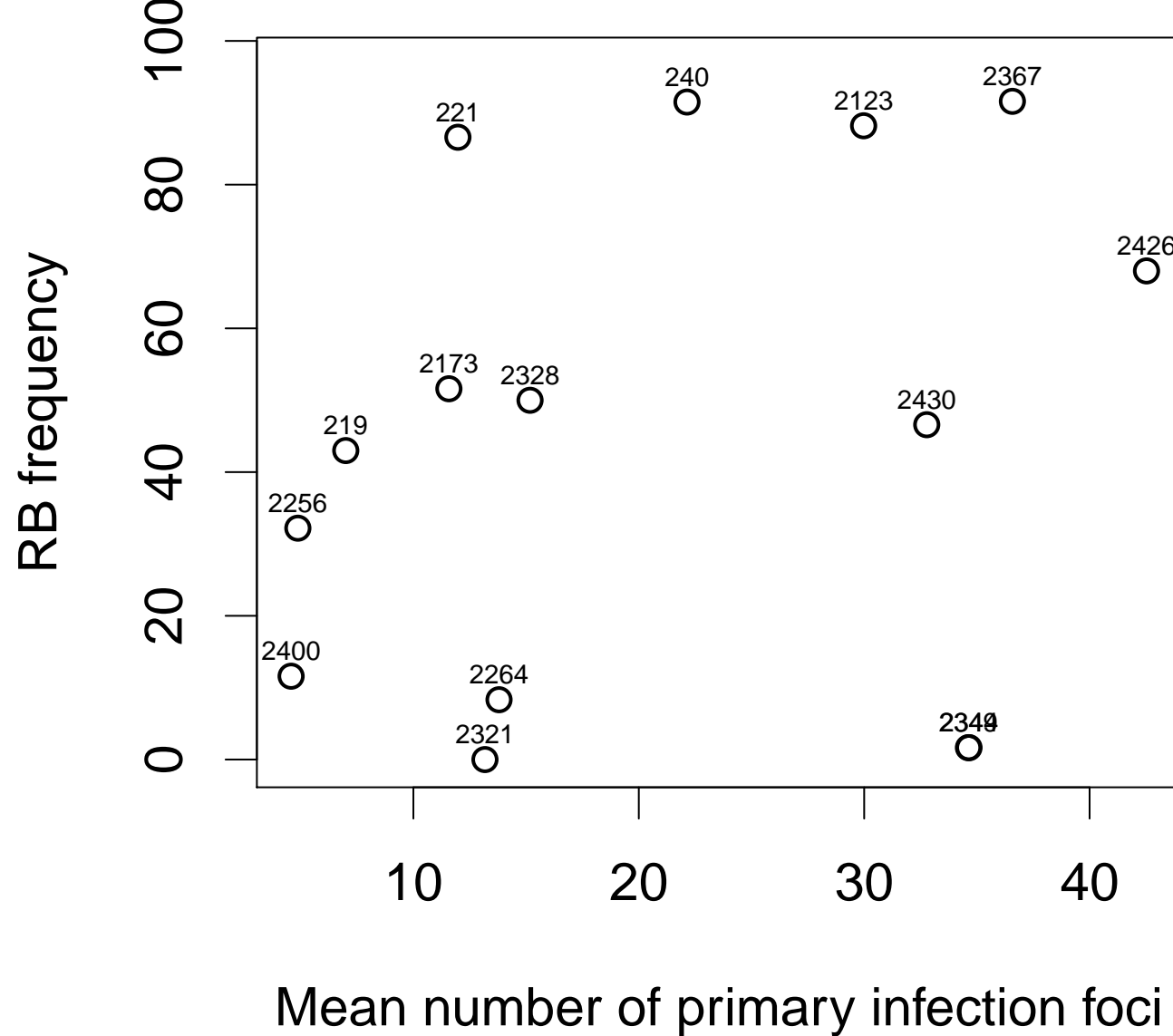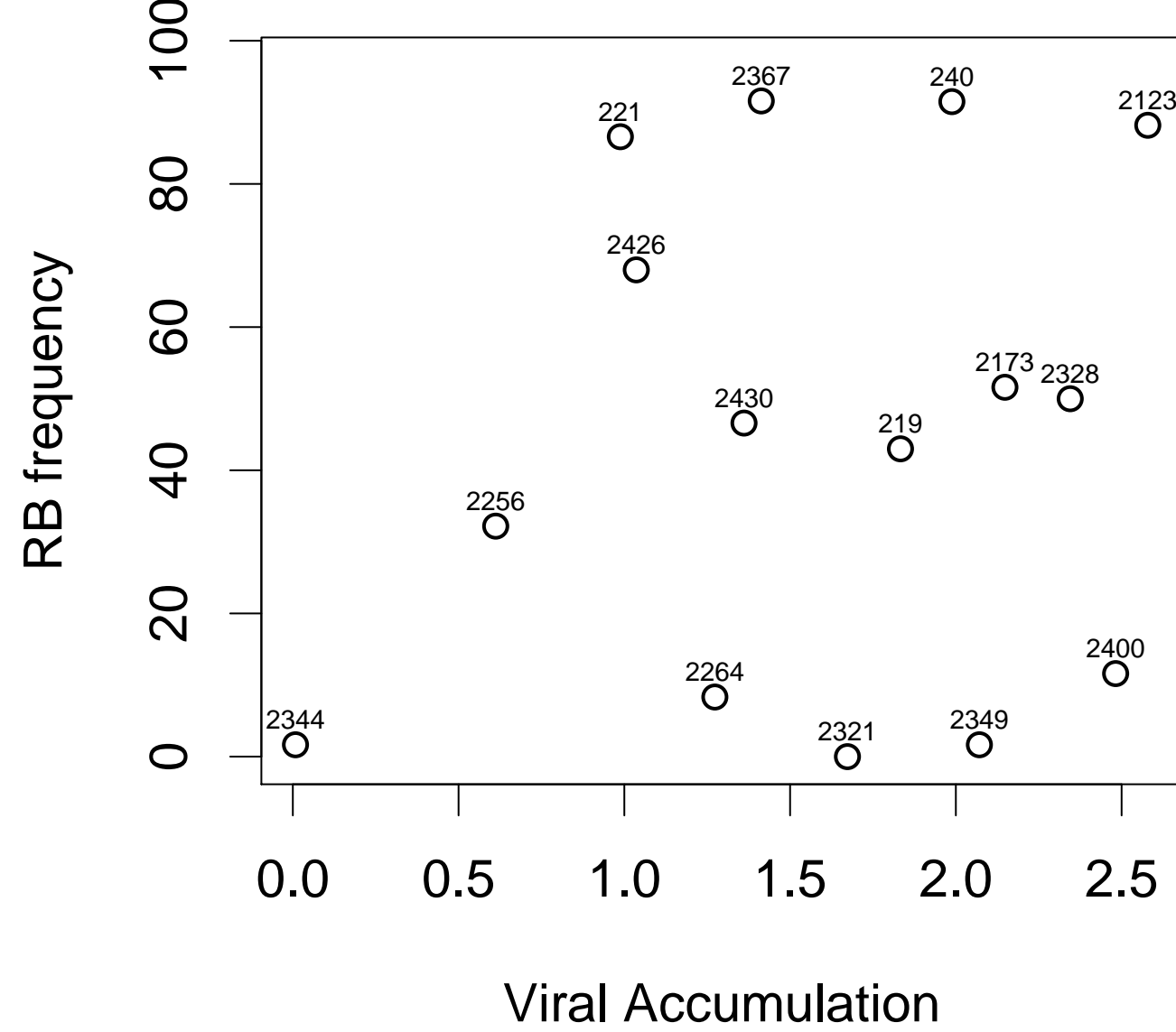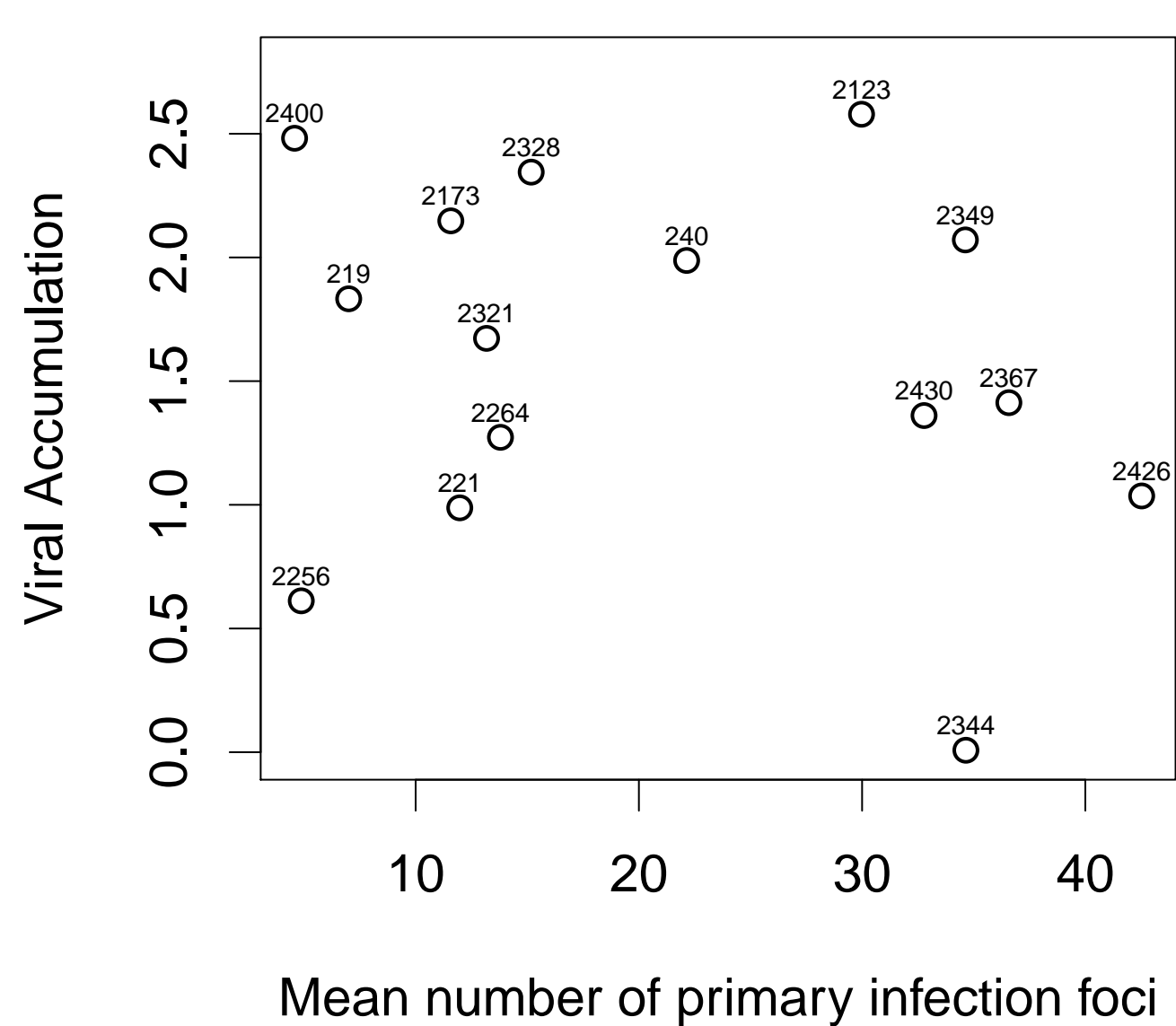

Supplement: S1 Fig — Pepper genotypes are represented as points, with their nomenclature (DH line number) given above each point. We estimated the mean number of primary infection foci for the 15 DH lines with the Potato virus Y (PVY, genus Potyvirus) variant K, carrying a green fluorescent marker (green fluorescent protein, GFP) [34]. The resistance-breakdown (RB) frequency and the relative viral accumulation were estimated by Quenouille et al. [12]. The RB frequency corresponds to the percentage of infected plants when inoculated with an avirulent variant regarding the allele of resistance pvr23, carried by all DH lines. The relative viral accumulation, or relative viral concentration, was measured by double antibody sandwich enzyme-linked immunosorbent assay (DAS-ELISA). (PDF) [file ppat.1006702.s004.pdf]

■ Variant G ■ Variant N ■ Variant K ■ Variant GK ■ Variant KN

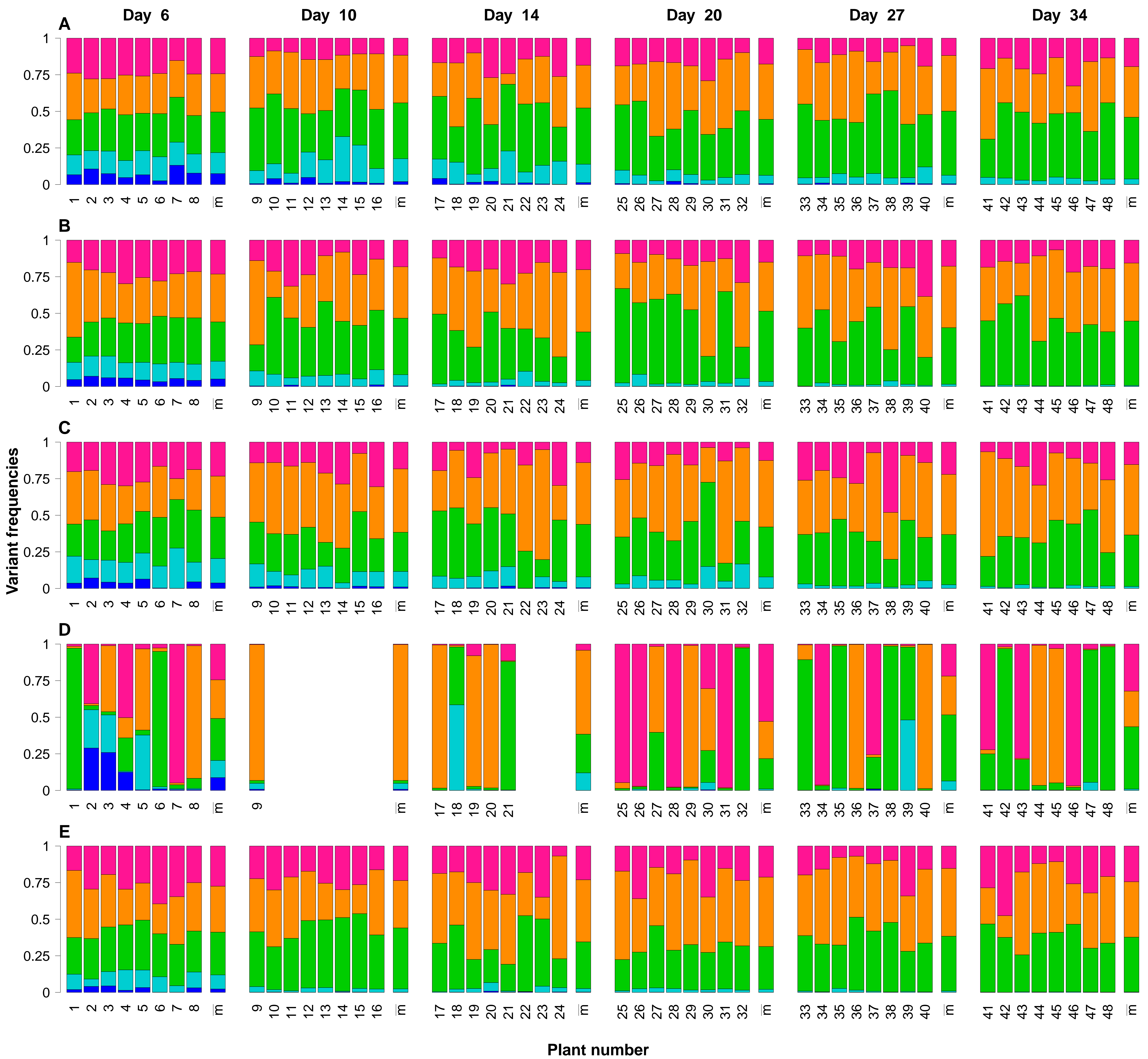

Supplement: S2 Fig — Each line of bar plots represents the dynamics of virus variants in a single DH line over time: (A) 221, (B) 2123, (C) 2173, (D) 2256 and (E) 2264. Within each bar plot, the frequencies of the five variants (see top of the figure for the color code) in each infected plant sample are represented by single bars (labeled from 1 to 48). The missing bars correspond to plant samples for which no viruses were detected. The last bar indicates the mean viral composition in the infected plants. Each individual bar plot corresponds to a single sampling date, indicated at the top of each column of barplots. (PDF) [file ppat.1006702.s005.pdf]

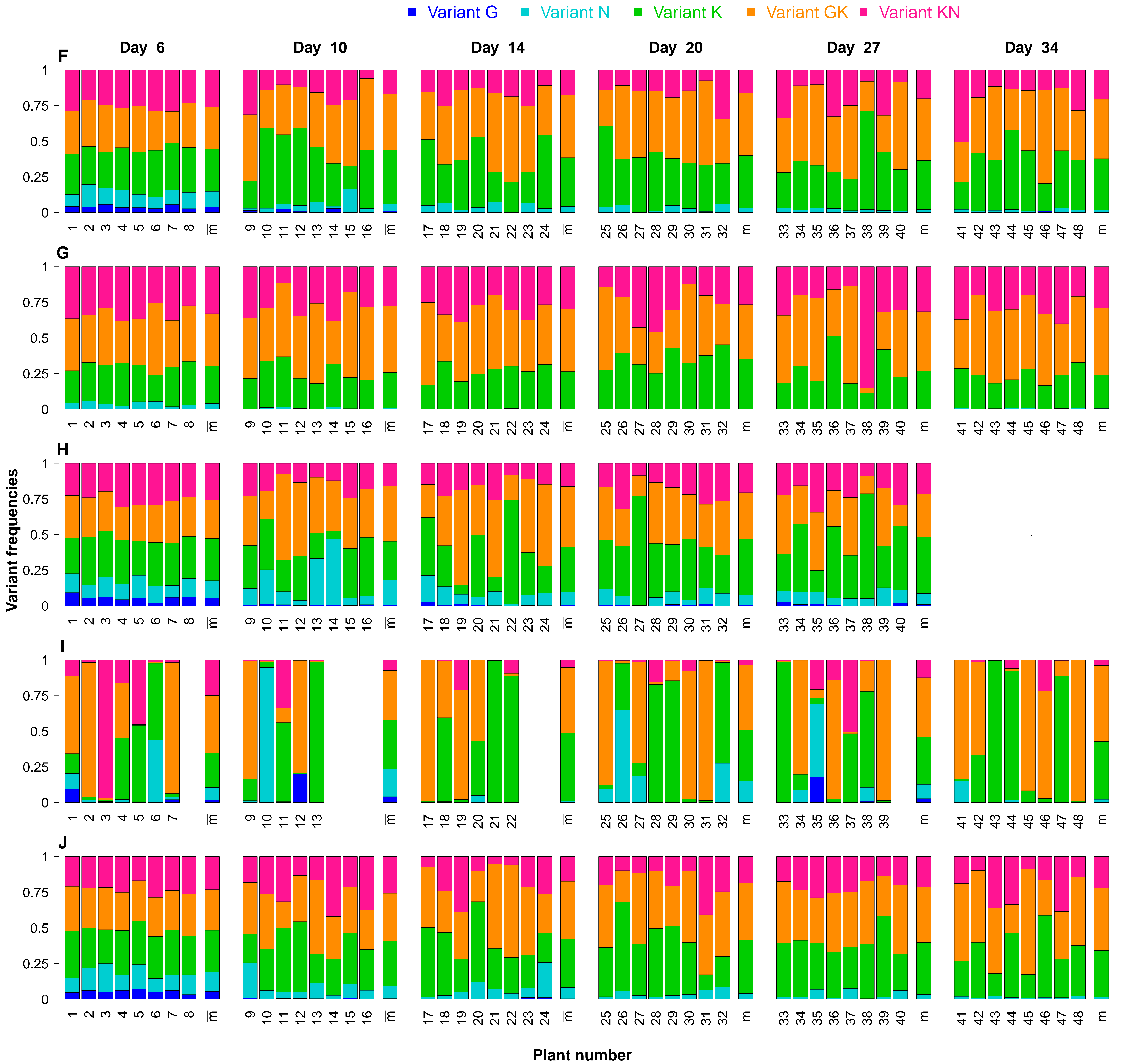

Supplement: S3 Fig — Each line of bar plots represents the dynamics of virus variants in a single DH line over time: (F) 2328, (G) 2349, (H) 2367, (I) 2400 and (J) 2426. Within each bar plot, the frequencies of the five variants (see top of the figure for the color code) in each infected plant sample are represented by single bars (labeled from 1 to 48). The missing bars correspond to plant samples for which no viruses were detected. The last bar indicates the mean viral composition in the infected plants. Each individual bar plot corresponds to a single sampling date, indicated at the top of each column of barplots. (PDF) [file ppat.1006702.s006.pdf]

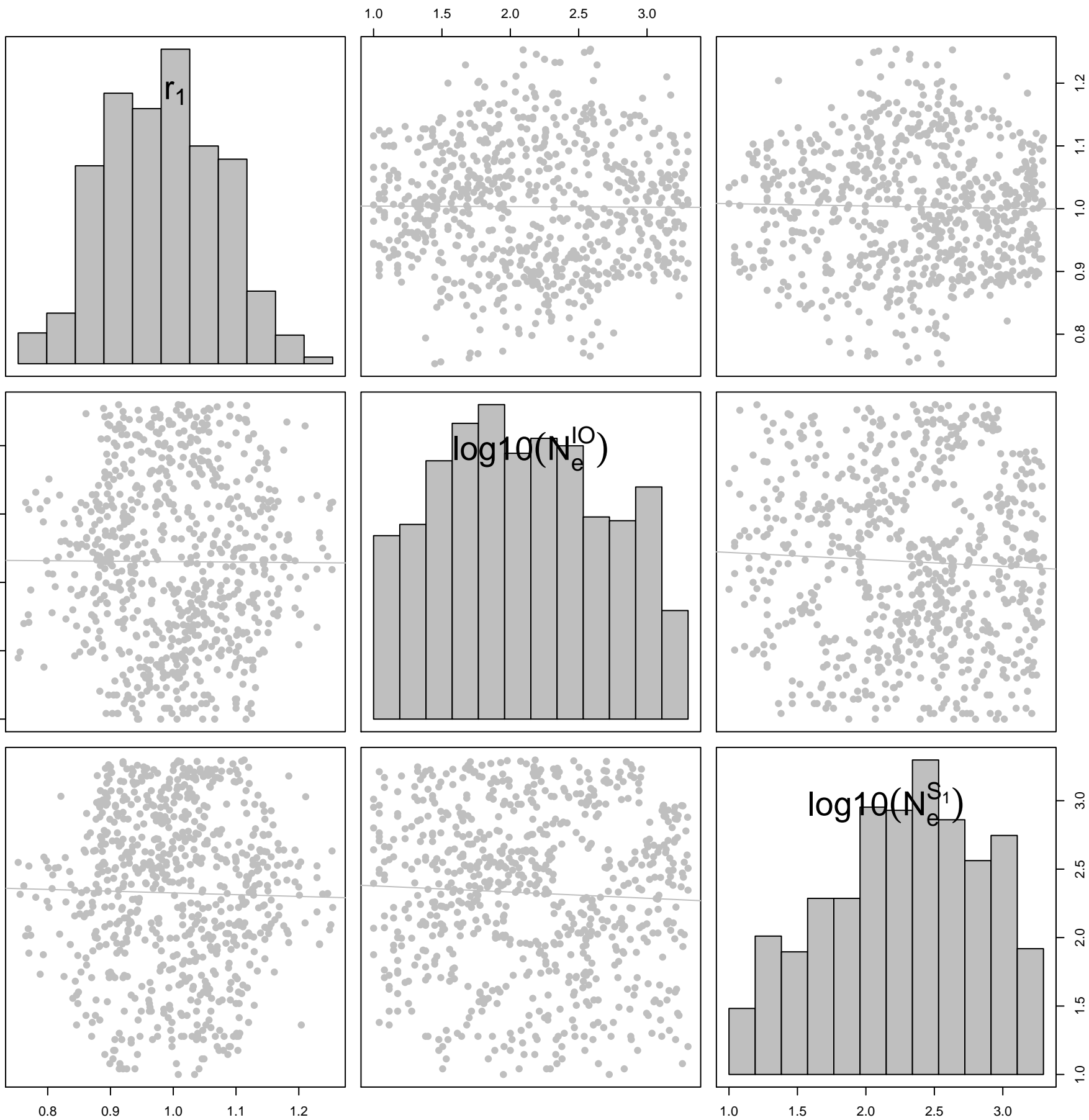

Supplement: S4 Fig — In the diagonal, each histogram represents the distribution of input parameters r1 (intrinsic rate of increase of variant 1), NeIO (effective population size in the inoculated organ) and NeS1 (effective population size at the onset of the systemic infection) used to simulate the 750 datasets. Off-diagonal scatter plots are two by two combinations of parameters. (PDF) [file ppat.1006702.s007.pdf]
