# Supplementary material for: Estimating virus effective population size and selection without neutral markers
Source: PLoS Pathog. 2017 Nov 20;13(11):e1006702. doi: 10.1371/journal.ppat.1006702 (PMC5720836; doi:10.1371/journal.ppat.1006702)
Supplement: S1 Table — The forward (Fwd.) primer sequence was the same for all amplifications and was bound to the sequence tag, just after it. Its binding site corresponds to positions 5971 to 5990 of PVY isolate SON41p (accession number AJ439544). The binding site of the reverse (Rev.) primer sequence corresponds to positions 6095 to 6114 of PVY isolate SON41p. RT-PCR amplifications were done according to the following profile: 1h at 42°C, 10 min at 95°C, 35 times the following sequence (45s at 95°C, 30s at 50°C and 20s at 72°C) and finally 10 min at 72°C. (PDF) [file ppat.1006702.s008.pdf]

	Name	Polarity	Sequence for binding libraries	Tag sequence	Primer sequence
Fwd.	Tag 1	+	5'-CTTTCCCTACACGACGCTCTTCCGATCT	ACGAGTGCGT	AAGAGAATGTCTATGCTGAC-3'
	Tag 2	+	5'-CTTTCCCTACACGACGCTCTTCCGATCT	CATAGTAGTG	AAGAGAATGTCTATGCTGAC-3'
	Tag 3	+	5'-CTTTCCCTACACGACGCTCTTCCGATCT	GGTCTAGTAC	AAGAGAATGTCTATGCTGAC-3'
	Tag 4	+	5'-CTTTCCCTACACGACGCTCTTCCGATCT	TCATACGCGT	AAGAGAATGTCTATGCTGAC-3'
	Tag 5	+	5'-CTTTCCCTACACGACGCTCTTCCGATCT	CTACGCTCTA	AAGAGAATGTCTATGCTGAC-3'
	Tag 6	+	5'-CTTTCCCTACACGACGCTCTTCCGATCT	ATCGATAGAC	AAGAGAATGTCTATGCTGAC-3'
	Tag 7	+	5'-CTTTCCCTACACGACGCTCTTCCGATCT	GAGGCTCTAC	AAGAGAATGTCTATGCTGAC-3'
	Tag 8	+	5'-CTTTCCCTACACGACGCTCTTCCGATCT	TGCTGATATC	AAGAGAATGTCTATGCTGAC-3'
Rev.	REV	-	5'-GGAGTTCAGACGTGTGCTCTTCCGATCT		CAGACCAATCTTTCCTGAAG-3'
