# Supplementary material for: Estimating virus effective population size and selection without neutral markers
Source: PLoS Pathog. 2017 Nov 20;13(11):e1006702. doi: 10.1371/journal.ppat.1006702 (PMC5720836; doi:10.1371/journal.ppat.1006702)
Supplement: S3 Table — Virus variants are indexed as follows: (i) r1 virus variant G, (ii) r2 virus variant N, (iii) r3 virus variant K, (iv) r4 virus variant GK, (v) r5 virus variant KN. The 90% confidence intervals are calculated as ri^±1.645.σi^. (PDF) [file ppat.1006702.s010.pdf]

Table S3. Estimations of the relative intrinsic rates of increase of the virus variants for the 15 plant genotypes.

HD line	r_1 (q-5%)	r_1 (mean)	r_1 (q-95%)	r_2 (q-5%)	r_2 (mean)	r_2 (q-95%)	r_3 (q-5%)	r_3 (mean)	r_3 (q-95%)	r_4 (q-5%)	r_4 (mean)	r_4 (q-95%)	r_5 (q-5%)	r_5 (mean)	r_5 (q-95%)
**2123**	0.7386	0.7425	0.7463	0.9630	0.9645	0.966	1.1113	1.1124	1.1135	1.1145	1.1156	1.1167	1.0636	1.0647	1.0659
**2173**	0.7529	0.7568	0.7608	0.9943	0.9956	0.9969	1.0872	1.0883	1.0894	1.1072	1.1083	1.1094	1.0496	1.0507	1.0518
**219**	0.9019	0.9032	0.9045	1.0358	1.0363	1.0368	1.0357	1.0362	1.0367	1.049	1.0495	1.0500	0.9738	0.9745	0.9752
**221**	0.8306	0.8329	0.8351	0.9945	0.9953	0.9962	1.0707	1.0713	1.072	1.0728	1.0735	1.0742	1.026	1.0267	1.0274
**2256**	0.8238	0.8276	0.8314	0.9508	0.9524	0.9539	1.0832	1.0843	1.0854	1.072	1.0731	1.0742	1.0613	1.0624	1.0635
**2264**	0.6868	0.6922	0.6977	0.9413	0.9434	0.9455	1.1220	1.1235	1.1251	1.142	1.1436	1.1451	1.0955	1.097	1.0985
**2321**	0.4171	0.4350	0.4529	0.8756	0.8814	0.8873	1.2045	1.2095	1.2144	1.2367	1.2418	1.2469	1.227	1.232	1.2371
**2328**	0.7484	0.7526	0.7567	0.9657	0.9673	0.9689	1.0963	1.0975	1.0986	1.1139	1.1151	1.1163	1.0662	1.0674	1.0686
**2344**	0.5155	0.5278	0.5400	0.9818	0.9850	0.9883	1.148	1.1513	1.1545	1.1919	1.1952	1.1985	1.1373	1.1405	1.1437
**2349**	0.4241	0.4403	0.4564	0.8759	0.8809	0.8858	1.2063	1.2107	1.2152	1.2463	1.2509	1.2554	1.2126	1.2171	1.2215
**2367**	0.8088	0.8115	0.8141	0.9989	1.0005	1.0011	1.0698	1.0706	1.0715	1.0828	1.0837	1.0845	1.0331	1.034	1.0349
**240**	0.8471	0.8493	0.8516	0.9966	0.9976	0.9986	1.0565	1.0573	1.0581	1.0752	1.0759	1.0767	1.0188	1.0196	1.0205
**2400**	0.7628	0.7673	0.7719	1.0091	1.0105	1.0119	1.1011	1.1024	1.1036	1.1205	1.1218	1.1231	0.9964	0.9977	0.9991
**2426**	0.7460	0.7495	0.7531	0.9832	0.9844	0.9857	1.0913	1.0923	1.0933	1.1079	1.109	1.11	1.0635	1.0646	1.0656
**2430**	0.7541	0.7580	0.7618	0.9620	0.9635	0.9651	1.105	1.1062	1.1073	1.1028	1.104	1.1051	1.067	1.0681	1.0692
