# Supplementary material for: Estimating virus effective population size and selection without neutral markers
Source: PLoS Pathog. 2017 Nov 20;13(11):e1006702. doi: 10.1371/journal.ppat.1006702 (PMC5720836; doi:10.1371/journal.ppat.1006702)
Supplement: S4 Table — The posterior probabilities of the four models considered for the piecewise function describing the temporal variation of the effective population sizes during the time course of the experiment (models M1, M2, M3 and M4) are first indicated. The bold value corresponds to the model that is best supported by the data. The next columns indicate the estimation of the effective population sizes of the model selected and the extent of the 90% credibility intervals. (PDF) [file ppat.1006702.s011.pdf]

Table S4. Model selection and estimations of the effective population sizes for the 15 plant genotypes.

	Posterior probabilities				days 1 to 6			days 7 to 10			days 11 to 14			days 15 to 34		
HD line	model 1	model 2	model 3	model 4	Ne (q-5%)	Ne (mean)	Ne (q-95%)	Ne (q-5%)	Ne (mean)	Ne (q-95%)	Ne (q-5%)	Ne (mean)	Ne (q-95%)	Ne (q-5%)	Ne (mean)	Ne (q-95%)
2123	**0.44**	0.17	0.22	0.16	216	349	543	216	349	543	216	349	543	216	349	543
2173	0.02	0	0.02	**0.96**	96	248	620	98	459	1951	55	225	1432	403	1095	2310
219	0.14	0.26	**0.32**	0.28	10	13	19	11	42	505	11	42	505	31	276	1909
221	0.24	0.05	**0.43**	0.28	237	552	1213	188	461	1286	188	461	1286	616	1368	2366
2256	0.07	0.03	**0.52**	0.38	10	13	21	11	43	440	11	43	440	11	61	1146
2264	**0.69**	0.07	0.08	0.17	252	393	597	252	393	597	252	393	597	252	393	597
2321	0.00	0.21	0.04	**0.74**	776	1329	2135	11	20	44	17	148	1687	64	400	2025
2328	0.00	0.14	0.10	**0.76**	430	866	1679	60	135	315	135	590	2139	181	534	1711
2344	0.19	0.29	0.11	**0.41**	107	421	1440	51	201	1283	72	412	2009	278	908	2269
2349	**0.35**	0.14	0.30	0.21	276	462	749	276	462	749	276	462	749	276	462	749
2367	0.00	0.35	0.31	**0.35**	526	883	1451	43	80	156	92	441	1989	254	858	2204
240	0.00	0.21	**0.52**	0.27	978	1515	2260	127	213	353	127	213	353	307	959	2275
2400	**0.84**	0.07	0.05	0.04	11	18	31	11	18	31	11	18	31	11	18	31
2426	0.01	**0.50**	0.24	0.26	383	805	1593	122	235	448	122	235	448	122	235	448
2430	0.07	0.23	**0.47**	0.23	61	1130	1971	165	308	601	165	308	601	420	1077	2269
